# Preoperative prediction of MGMT promoter methylation in glioblastoma based on multiregional and multi-sequence MRI radiomics analysis

**DOI:** 10.1038/s41598-024-66653-2

**Published:** 2024-07-11

**Authors:** Lanqing Li, Feng Xiao, Shouchao Wang, Shengyu Kuang, Zhiqiang Li, Yahua Zhong, Dan Xu, Yuxiang Cai, Sirui Li, Jun Chen, Yaou Liu, Junjie Li, Huan Li, Haibo Xu

**Affiliations:** 1 Department of Radiology, Zhongnan Hospital of Wuhan University, Wuhan, China; 2 Department of Radiology, Sir Run Run Shaw Hospital (SRRSH) of School of Medicine, Zhejiang University, Hangzhou, Zhejiang China; 3 Department of Neurosurgery&Brain Glioma Center, Zhongnan Hospital of Wuhan University, Wuhan, China; 4 Department of Oncology, Zhongnan Hospital of Wuhan University, Wuhan, China; 5 Department of Nuclear Medicine, Zhongnan Hospital of Wuhan University, Wuhan, China; 6 Department of Pathology, Zhongnan Hospital of Wuhan University, Wuhan, China; 7 Wuhan GE Healthcare, Wuhan, China; 8 Department of Radiology, Beijing Tiantan Hospital, Capital Medical University, Beijing, China

**Keywords:** Cancer imaging, CNS cancer, Image processing, Machine learning, Predictive markers

## Abstract

O6-methylguanine-DNA methyltransferase (MGMT) has been demonstrated to be an important prognostic and predictive marker in glioblastoma (GBM). To establish a reliable radiomics model based on MRI data to predict the MGMT promoter methylation status of GBM. A total of 183 patients with glioblastoma were included in this retrospective study. The visually accessible Rembrandt images (VASARI) features were extracted for each patient, and a total of 14676 multi-region features were extracted from enhanced, necrotic, “non-enhanced, and edematous” areas on their multiparametric MRI. Twelve individual radiomics models were constructed based on the radiomics features from different subregions and different sequences. Four single-sequence models, three single-region models and the combined radiomics model combining all individual models were constructed. Finally, the predictive performance of adding clinical factors and VASARI characteristics was evaluated. The ComRad model combining all individual radiomics models exhibited the best performance in test set 1 and test set 2, with the area under the receiver operating characteristic curve (AUC) of 0.839 (0.709–0.963) and 0.739 (0.581–0.897), respectively. The results indicated that the radiomics model combining multi-region and multi-parametric MRI features has exhibited promising performance in predicting MGMT methylation status in GBM. The Modeling scheme that combining all individual radiomics models showed best performance among all constructed moels.

## Introduction

Glioblastoma (GBM) is the most common and lethal malignant primary brain tumor in adults^1^, with an incidence of 50.1% of all malignant brain tumors and a 5 year survival rate of only 6.9%^2^. Standard treatment for glioblastoma includes surgery, postoperative radiotherapy, and temozolomide (TMZ) adjuvant chemotherapy. Despite multimodal treatment, patients continue to have a poor prognosis with a median survival of approximately 12–15 months^3^. Intra-tumor heterogeneity and mutational spectrum are important factors affecting survival outcomes^4^. MGMT is a DNA repair enzyme that antagonizes the alkylating effect of the alkylating agent TMZ on the O6 guanine group, reducing the cytotoxic effect of the tumor cells and rendering the cells resistant to the drug. Gene silencing due to methylated MGMT results in a reduction of this antagonism and an increase in tumor sensitivity to chemotherapy^5^. Patients with MGMT methylation have longer progression-free survival compared to those without MGMT methylation^6^. Therefore, the detection of MGMT promoter methylation is important for the patients in evaluating their response to chemotherapy and the improvement of the prognosis^7^.

Histological evaluation after surgery or biopsy is currently the gold standard for genetic and molecular testing of tumors; however, these must be obtained invasively and have the disadvantages of high cost, long time to obtain results, risk of complications, and incomplete sampling due to tumor heterogeneity^8^. Therefore, it is necessary to develop a preoperative non-invasive detection method for MGMT promoter methylation status.

MRI is the most reliable imaging modality for the diagnosis of glioma, which can noninvasively provide a large amount of 3D tumor information throughout the heterogeneous tumor region for predicting the genomic profile, treatment response, and prognosis of glioma. Previous studies have shown that MGMT status is significantly correlated with certain imaging features on MR Images, such as tumor location, enhancement mode, necrotic tumor volume, and T2 / FLAIR hyperintense volume^9–13^. The emerging field of radiomics associates MRI radiological features with gene expression profiles as a non-invasive and reproducible alternative to histological testing^11,14,15^. Radiomics is the process of extracting high-throughput data features from quantitative image data, analyzing and modeling, aiming to improve the accuracy of computer-aided medical diagnosis, prognosis and prediction, and help to assist medical decision-making^16,17^. In the field of glioma research, some recent studies have shown that radiomics has been widely used in predicting molecular subtypes^18,19^, survival rate and survival stratification^20–22^, grading^23^, differential diagnosis^24,25^, identification of tumor invasion boundaries^26,27^, and differentiation between tumor recurrence and brain radiation injury^28,29^.

Due to the intratumor heterogeneity of glioblastoma^30^, the radiomics model composed of features from multiple tumor subregions can more precisely explore the tumor microenvironment compared with the radiomics model composed of features from the whole tumor region, and shows reliable predictive value. Multi-sequence MRI and multi-subregion radiomics have been widely recognized in the prediction of molecular subtypes of tumors^31^.

The aim of this study was to retrospectively collect MRI data, and to develop a radiomics model by incorporating imaging features from multiple tumor subregions on multi-sequence MRI to predict preoperative MGMT methylation status in patients with glioblastoma.

## Results

### Patients

As shown in Fig. S1, a total of 183 pathologically confirmed glioblastoma patients (our hospital: 100, TCIA: 43 and Tiantan hospital: 40) were enrolled in this study. Tables 1, 2 showed the demographics and semantic imaging feature of the patients, and we found except the gender and the deep white matter invasion, there were no significant differences between the MGMT unmethy and the MGMT methy groups for the other characteristics.

**Table 1:** Patients demographics and semantic imaging feature for training set and test set 1.

Characteristic	Training set (100)		*p*-value	Test set 1 (43)		*p*-value
	MGMT unmethy. (n = 54)	MGMT methy. (n = 46)		MGMT unmethy. (n = 23)	MGMT methy. (n = 20)	
Age	59.04 ± 8.90	59.64 ± 12.16	0.783	62.39 ± 11.16	58.33 ± 10.48	0.226
Gender						
Female	19	24	0.032	6	9	0.329
Male	35	22		17	11	
Location						
Frontal	12	15	0.766	6	4	0.948
Temporal	15	11		7	5	
Parietal	6	4		3	5	
Occipital	6	7		2	1	
Insular	1	0		1	1	
Others	14	9		4	4	
Centerside						
Right	23	21	0.671	6	8	0.418
Center/Bilateral	12	7		3	4	
Left	19	18		14	8	
Enhancement						
None	0	0	0.791	0	0	0.711
Mild	10	7		4	5	
Marked	44	39		19	15	
Cysts						
No	42	33	0.643	18	14	0.788
Yes	12	13		5	6	
Multifocal/Multicentric						
Focal	41	39	0.351	19	13	0.148
Multifocal	8	6		3	7	
Multicentric	5	1		1	0	
T1/FLAIR Ratio						
Expansive	31	25	0.264	14	10	0.764
Mixed	21	17		8	9	
Infiltrative	2	4		1	1	
Enhancing.Margin.Thick						
< 3 mm	21	21	0.357	8	12	0.196
> 3 mm	28	24		14	7	
Solid	5	1		1	1	
Enhancing.Margin						
Well defined	48	40	0.769	22	17	0.324
Poorly defined	6	6		1	3	
Edema.Proportion						
None	3	4	0.859	1	2	0.806
< 33%	22	19		11	9	
> 33%	29	23		11	9	
Hemorrhage						
No	43	33	0.493	16	13	1.000
Yes	11	13		7	7	
Pial.Invasion						
No	34	22	0.052	9	11	0.463
Yes	20	24		14	9	
Ependymal.Extension						
No	32	28	1.000	12	13	0.589
Yes	22	18		11	7	
Cortical.Involvement						
No	21	14	0.501	5	6	0.728
Yes	33	32		18	14	
Deep.WM.Invasion						
No	19	24	0.032	15	8	0178
Yes	35	22		8	12	
nCET_Mid						
No	37	36	0.386	19	15	0.711
Yes	17	10		4	5	
CET_Mid						
No	48	41	1.000	21	18	1.000
Yes	6	5		2	2	
Satellites						
No	35	33	0.600	14	14	0.760
Yes	19	13		9	6

**Table 2:** Patients demographics and semantic imaging feature for test set 2.

Characteristic	Test set 2 (40)		*p*-value
	MGMT unmethy. (n = 26)	MGMT methy. (n = 14)	
Age	51.54 ± 10.44	55.43 ± 11.06	0.289
Gender			
Female	11	9	0.320
Male	15	5	
Deep.WM.Invasion			
No	12	11	0.092
Yes	14	3	

*MGMT unmethy* MGMT unmethylated, *MGMT methy* MGMT methylated, *Deep.WM.Invasion* deep white matter invasion; *nCET_Mid* non-contrast-enhancing tumour crosses midline, *CET_Mid* contrast-enhancing tumour crosses midline.

### Images analysis and model construction

**Figure 1:**
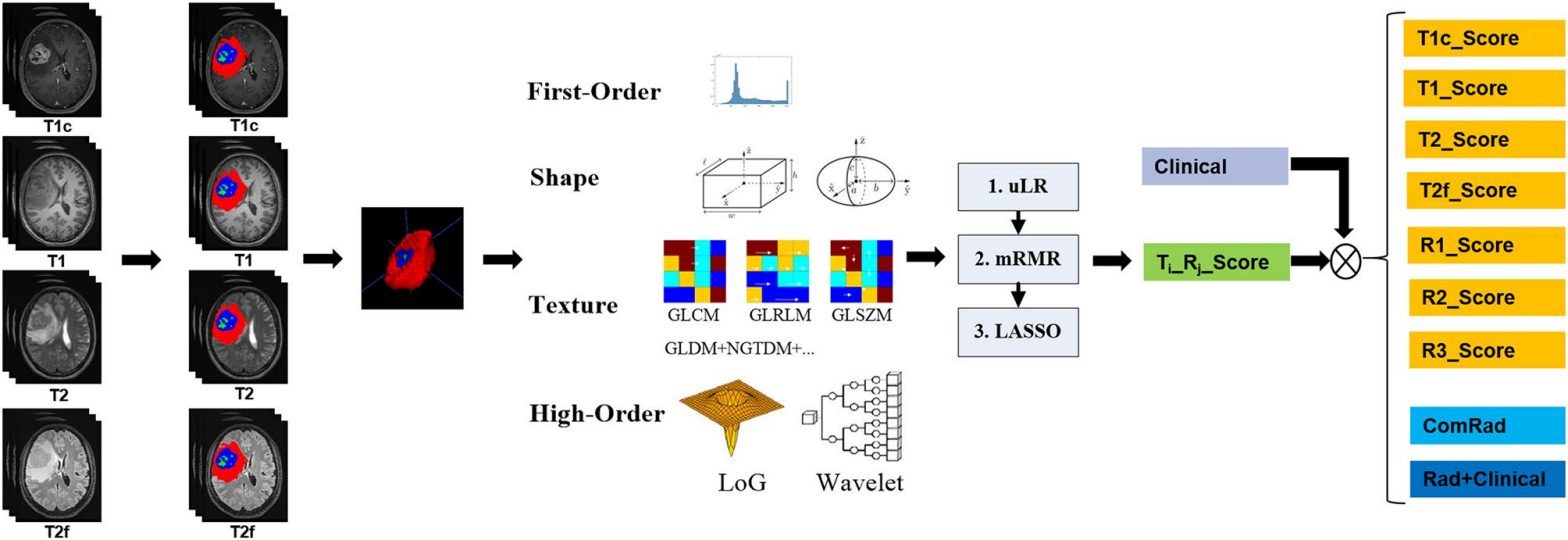
The data flowchart of this study. From the left to right, the four steps of data analysis in this study are: image registration and segmentation, features extraction, feature reduction and machine learning modeling, and combined modeling. In this figure, T_*i*__R_*j*__score represented the model constructed using the *i*th modal images (T1c, T1, T2 or T2f.) with the *j*th ROI (NE_EDEMA, NEC or CE).

**Figure 2:**
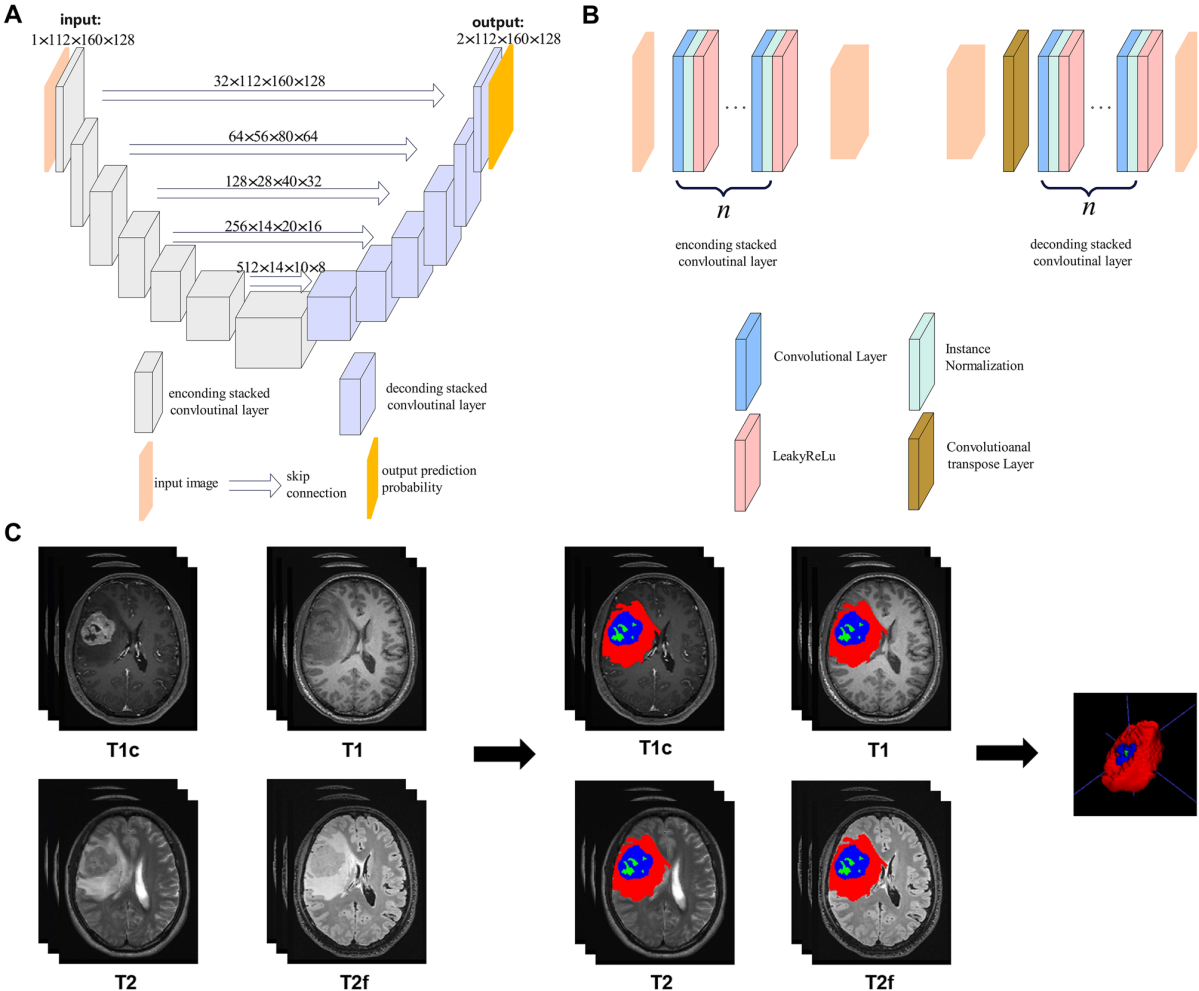
Gliomas segmentation using an 3D U-shape convolutional neural network. (**A**) the model architecture of the 3D U-CNN; (**B**) the detailed construction of the model; (**C**) the gliomas segmentation results of the 3D U-CNN, three ROIs were deliminated for each gliomas and showed in different colors: Red (NE_EDEMA), Green (NEC) and Blue (CE).

As shown in Fig. 1, after the registration and preprocessing, all the images of the four modals for each patient were inputted into the 3D Unet model, and were deliminated into three ROIs with three colors (Fig. 2C), which represented the gliomas area of the NE_EDEMA, NEC and CE respectively. For each patient, 1223 radiomics features were extracted for each ROI in each image modal. Based on these 12*1223 radiomics features, we built 12 individual radiomics models: the feature number retained using uLR for different individual model was not the same, but the features numbers retained from the mRMR selection were set as a same value of 50. And the details of LASSO modeling for the 12 individual models were shown in Figs. S3–S6. Tables S1–S12 showed the final selected features and their corresponding weights for the 12 individual models. Table S13 showed the Clinical model constructed using the screened two risk factors: the gender and the deep white matter invasion. Table S14 showed the detailed ROC-related evaluation metrics (including AUC, Accuracy, Sensitivity and Specificity) for the 12 individual models.

Finally, nine combination models were constructed using the strategy shown in Table 3. The features retained for the nine combination models, and the model coefficients were shown in Tables S15–S23, respectively. The coefficients for each feature showed in the model construction represented the contribution of each feature to the corresponding model.

**Table 3:** Construction details for different combined models.

Models	Variables	Methods
Ti_Rj_Score	Radiomics features in *i*th ROI in the T_j_ image modal	uLR + mRMR + LASSO
T1cScore	T1c_R1_Score + T1c_R2_Score + T1c_R3_Score	LR
T1Score	T1_R1_Score + T1_R2_Score + T1_R3_Score	LR
T2Score	T2_R1_Score + T2_R2_Score + T2_R3_Score	LR
T2fScore	T2f_R1_Score + T2f_R2_Score + T2f_R3_Score	LR
ROI1Score	T1c_R1_Score + T1_R1_Score + T2_R1_Score + T2f_R1_Score	LR
ROI2Score	T1c_R2_Score + T1_R2_Score + T2_R2_Score + T2f_R2_Score	LR
ROI3Score	T1c_R3_Score + T1_R3_Score + T2_R3_Score + T2f_R3_Score	LR
CombineRad	T1c_R1_Score + T1c_R2_Score + T1c_R3_Score + T1_R1_Score + T1_R2_Score + T1_R3_Score + T2_R1_Score + T2_R2_Score + T2_R3_Score + T2f_R1_Score + T2f_R2_Score + T2f_R3_Score	LR
Clinical	Gender + Deep.White.Matter.Invasion	LR
Rad + Clinical	Gender + Deep.White.Matter.Invasion + CombRadScore	LR

T1:T1-weighted; T1c:T1-weighted gadolinium contrast-enhanced; T2: T2-weighted; T2f.: T2-weighted FLAIR. ROI1: the “non-enhancing and edema” region (NE_EDEMA), which was defined as FLAIR hyperintense abnormality, excluding the contrast-enhancing and necrotic tumor region; ROI2: the necrosis region (NEC); ROI3: the contrast enhancement region (CE).

*uLR* univariate logistic regression, *mRMR* maximum relevance minimum redundancy, *LASSO* least absolute shrinkage and selection operator, *LR* logistic regression.

**Figure 3:**
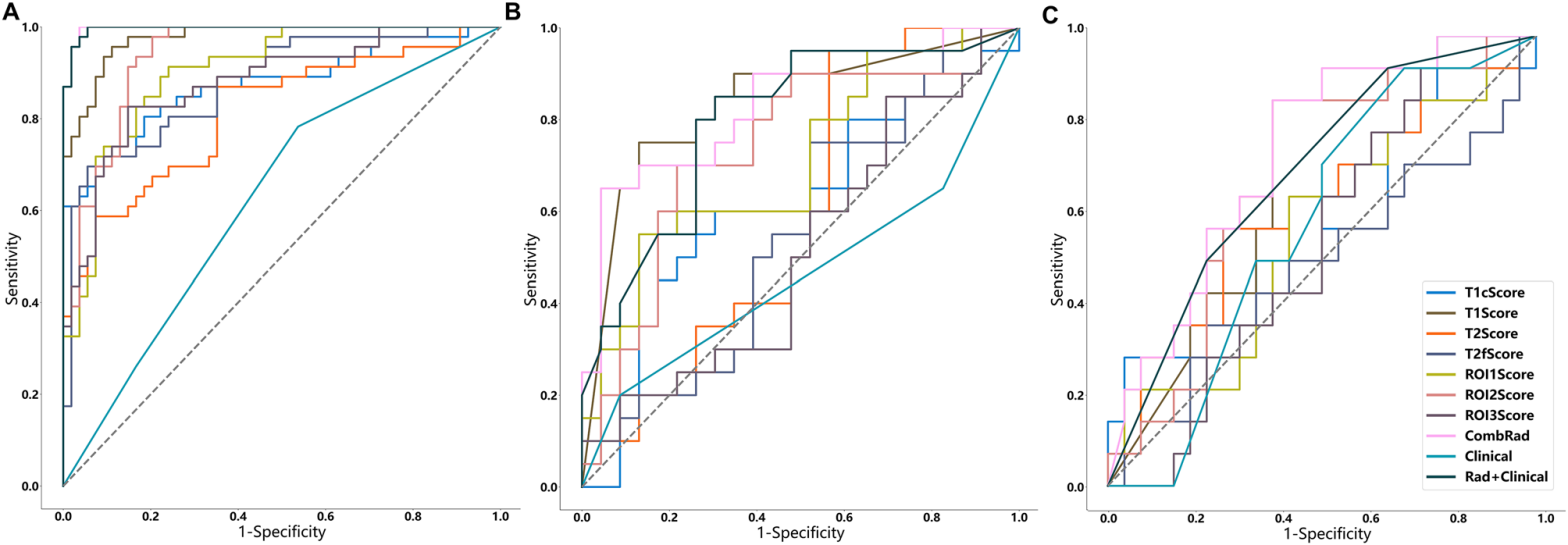
ROC used to assess the prediction accuracy of the constructed models. All models were evaluated in the training set (**A**), test set 1 (**B**) and test set 2 (**C**). The diagonal dashed line represented the AUC value of 0.5, which means a completely random prediction.

Model validation and comparison were performed using ROC analysis. Figure 3 shows the ROC curves of all constructed combination models, and Table 4 provides their detailed ROC-related metrics, including AUC, Sensitivity, and Specificity. The ComRad model achieved the highest performance with an AUC of 0.839 (95% CI 0.709–0.963) on test set 1 and 0.739 (95% CI 0.581–0.897) on test set 2. These results indicate that the multi-sequence, multi-region radiomics model significantly outperforms models based on single sequences or single regions.

**Table 4:** Detailed ROC-related metrics for all constructed models.

Models		Strategy 1			
		AUC(95%CI)	Accuracy	Sensitivity	Specificity
T1cScore	Train	0.873 (0.8–0.946)	0.82	0.696	0.926
	Test1	0.609 (0.431–0.786)	0.651	0.6	0.696
	Test2	0.566 (0.366–0.765)	0.725	0.286	0.962
T1Score	Train	0.976 (0.954–0.998)	0.92	0.957	0.889
	Test1	0.836 (0.717–0.962)	0.814	0.65	0.957
	Test2	0.662 (0.483–0.841)	0.7	0.857	0.615
T2Score	Train	0.813 (0.728–0.899)	0.75	0.87	0.648
	Test1	0.598 (0.422–0.774)	0.674	0.95	0.435
	Test2	0.615 (0.421–0.81)	0.675	0.571	0.731
T2fScore	Train	0.878 (0.81–0.945)	0.83	0.696	0.944
	Test1	0.559 (0.382–0.735)	0.605	0.75	0.478
	Test2	0.495 (0.292–0.697)	0.625	0.357	0.769
R1Score	Train	0.902 (0.843–0.96)	0.83	0.913	0.759
	Test1	0.709 (0.55–0.867)	0.721	0.55	0.87
	Test2	0.569 (0.378–0.76)	0.6	0.643	0.577
R2Score	Train	0.938 (0.893–0.982)	0.88	0.978	0.796
	Test1	0.737 (0.58–0.894)	0.744	0.7	0.783
	Test2	0.684 (0.511–0.857)	0.7	0.857	0.615
R3Score	Train	0.878 (0.81–0.946)	0.84	0.826	0.852
	Test1	0.485 (0.306–0.664)	0.581	0.7	0.478
	Test2	0.544 (0.364–0.724)	0.5	1	0.231
CombRad	Train	0.997 (0.991–1)	0.98	1	0.963
	Test1	0.839 (0.709–0.963)	0.814	0.75	0.87
	Test2	0.739 (0.581–0.897)	0.7	0.857	0.615
Clinical	Train	0.628 (0.522–0.734)	0.61	0.783	0.463
	Test1	0.52 (0.339–0.7)	0.605	0.45	0.739
	Test2	0.58 (0.403–0.756)	0.525	0.929	0.308
Rad + Clinical	Train	0.996 (0.99–1)	0.97	1	0.944
	Test1	0.802 (0.666–0.938)	0.767	0.85	0.696
	Test2	0.694 (0.538–0.85)	0.55	0.929	0.346

Youden criterion: The best operating point of the ROC was chosen at the point whose Youden index (Sensitivity + Specificity-1) is maximal.

### Models’ validation and comparison

**Figure 4:**
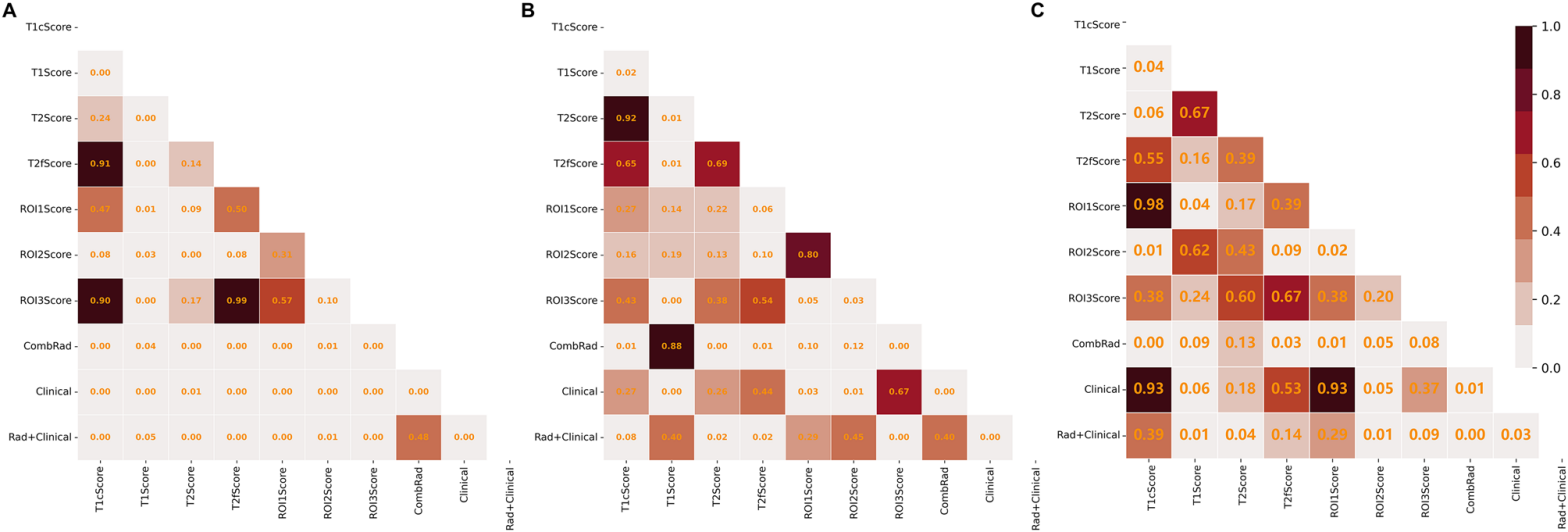
Delong test results (*p*-value maps) between different models. Each block in the map showed the p-value of Delong test between corresponding two models in the training set (**A**), test set 1 (**B**) and test set 2 (**C**). The closer the block color is to light white, the smaller the p-value and the more significant of performance difference between the models.

Figure 3 showed the ROC of all constructed combination models, and Table 4 showed their detailed ROC-related metrics: AUC, Sensitivity and Specificity. P-value shown in Fig. 4 depicted whether the prediction accuracy difference between two models. Combined with Figs. 3, 4 and Table 4, we could find that:

- (1) Among the combination models constructed on four image modals, the model constructed based on T1 images showed the best prediction performance (AUC = 0.836 in test set 1 and 0.662 in test set 2) and the other three models showed comparable performance with no significant difference. Except the T2 model in test set 2, the prediction accuracy of T1 model was all significantly better than that of the other three models in two test sets (*p* < 0.05).
- (2) Among the combination models constructed on three ROIs, the model constructed based on ROI1 and ROI2 showed significant better prediction performance than that on the ROI3 (p < 0.05), and the prediction difference between the models constructed based on ROI1 and ROI2 was not significant.
- (3) Among all models, the combination of all individual models (ComRad) showed the best prediction performance (AUC = 0.839 in test set 1 and 0.739 in test set 2).
- (4) Compared to the ComRad model, the incorporation of clinical risk factors (Clinical + Rad) did not improve the model performance, and degrade its performance instead, for both two test sets. (AUC = 0.802 in test set 1 and 0.694 in test set 2).

### Clinical use and explanation

**Figure 5:**
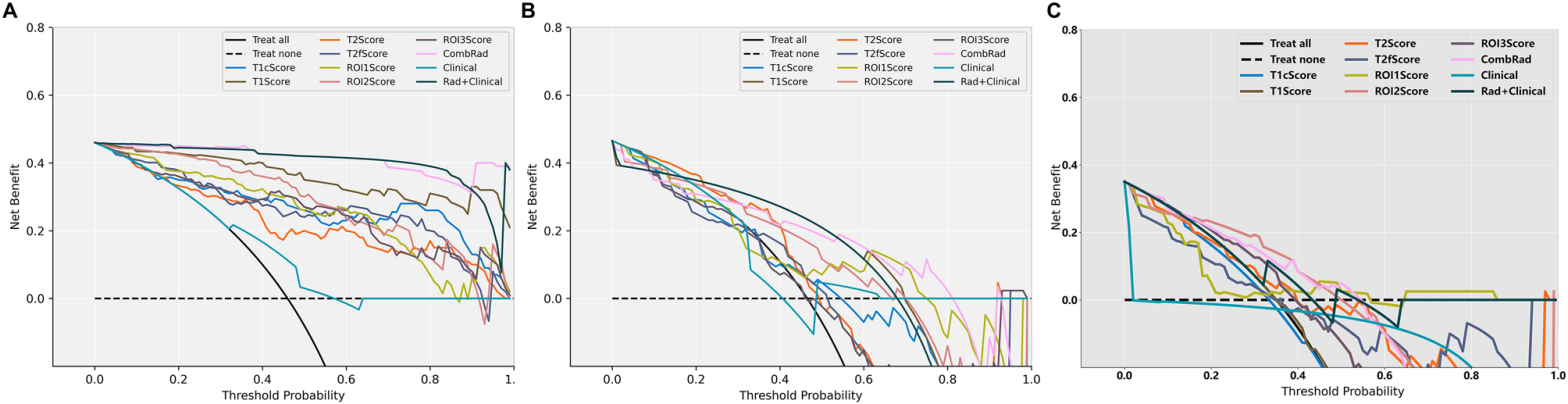
Decision curve used to assess the clinical net benefits when the constructed models were used. All models were evaluated in both training set (**A**), test set 1 (**B**) and test set 2 (**C**). Treat-all strategy: The net benefit in the condition that all patients were treated as Positive; Treat-none: All patients were treated as Negative.

Figure 5 showed the net benefit of the combination models obtained in their clinical use. The comparison results revealed:

- (1) For the test set 1, the ComRad, Clinical + Rad, and the combination models based on T1, ROI1 and ROI2 (T1Score, R1Score and R2Score), could bring more benefits than the "treat all" or "treat none" strategies was used for most of the risk thresholds.
- (2) The use of T1Score, T1Score, Clinical + Rad, ComRad or ROI1Score models could obtain more net benefit than that of the others at different risk thresholds range. (about 0.0–0.22: T2Score; 0.22–0.55: T1Score/Clinical + Rad; 0.55–0.9: ComRad or ROI1Score) in test set1 1.
- (3) For test set 2, except the ROI1, T2fand Clinical models, all other constructed models could bring more benefits than the “treat all” or “treat none” strategies when risk thresholds larger than about 0.35.

## Discussion

In this study, multiple conventional MRI sequences (T1WI, T2WI, T2-FLAIR, CE-T1WI) were used to perform radiomics analysis based on imaging features provided by multiple tumor subregions for preoperative prediction of MGMT promoter methylation in glioblastoma. Compared with the model using only a single MRI sequence or the whole tumor region, the final radiomics model integrating multiple MRI sequences and multiple regions was found to be more accurate in identifying MGMT methylation.

Several previous studies have predicted MGMT methylation with visually assessed imaging features, tumor volume, texture features, and VASARI features^13,32,33^. These features may not fully reflect imaging information, and the radiomics model constructed by these features has limitations in predictive performance. Recent studies have shown that significant levels of intra-tumoral microenvironment and anatomical multiregional heterogeneity exist in GBM^30,34^. Multi-habitat radiological features, including necrosis, edema, and enhancement, can reflect tumor heterogeneity in different regions. The molecular subtype prediction and prognostic value of multi-region radiomics has been demonstrated^4,35^. Based on 14676 features from multiple tumor subregions in multi-sequence MRI, we established four single-sequence models, three subregion models and combined models, which could more comprehensively characterize the changes of the tumor microenvironment. We validated these models on two test sets and obtained relatively consistent results, illustrating the versatility and robustness of the prediction model.

Previous studies have constructed a radiomics model with 6 fully correlated features from 6 sub-regions and 4 image modalities, with an AUC of 0.88 and an accuracy of 80%^36^。Wei et al.^37^ combined the radiomics features of the tumor and peritumoral edema area on the three imaging sequences to predict the MGMT status of grade II-IV astrocytoma, and the model fused with radiomics features achieved the best performance. Our results showed that the combined multi-sequence and multi-subregion radiomics model achieved the best prediction performance, which was better than all individual radiomics models based on a single sequence or a single subregion, reflecting the results of previous studies. Yogananda et al.^38^ simultaneously single-label segmented tumors and predicted the presence of MGMT promoter methylation on 3D-dense-UNets using only T2WI images, and the average cross-validation accuracy and AUC reached 0.95 and 0.93. Although these studies achieved higher performance, with AUCs ranging from 0.88 to 0.925, they used only private or TCIA data sets with small sample sizes and some lacked independent external validation, which most likely contributed to the increased risk of overfitting.

In the research of predicting MGMT methylation status, the BRaTS dataset is commonly used as a benchmark for training and validating various predictive models. In the rankings of the BRaTS 2021 challenge^39^, the first place model had a predictive power of 0.62. Since then, studies conducted by researchers such as Kim et al.^40^, Capuozzo et al.^41^, Faghani et al.^42^, and Saxena et al.^43^ have provided valuable baseline results, with the best AUC and accuracy generally ranging between 0.56 and 0.75. However, the performance of their models are generally lower than the performance achieved by our models using multiparametric MRI data, which exceed an AUC and accuracy of 0.80. Given the large sample size and significant validation potential of the BRaTS database, we plan to incorporate it into our future research, which will further validate and optimize our predictive model and enhance its generalizability and accuracy across broader datasets.

The results also showed that among the “single-sequence models”, the T1WI model showed the best predictive performance. This is partially consistent with the study by He et al.^44^, which collected five MRI sequences of 81 glioma patients for radiomics analysis and found that the AUC of the T1WI model was the highest among all single-sequence models in differentiating MGMT status. However, Huang et al.^45^ used multiple logistic regression analysis to construct the MGMT classification model, and pointed out that texture features of CE-T1WI accounted for the highest weight in the combined model. Another study^46^ showed that the shallow CNN model trained on T2-FLAIR images had better performance in distinguishing MGMT states. These conclusions are inconclusive and may be due to differences in image acquisition protocols and study procedures, among others. To improve the accuracy of the prediction results, future studies should be conducted using larger samples based on data from more clinical centers.

Han et al.^12^ showed that tumor necrosis was more likely to occur in GBM patients with MGMT promoter methylation. One study^44^ showed that MGMT methylated type had less edema than unmethylated type. Our results showed that among the “single subregion model”, the necrotic area model had the best prediction performance, and there was no significant difference between the necrotic area model and the "non-enhanced edema area" model, which was similar to previous studies. The radiophenotypes of different tumor subregions contribute different, complementary values in terms of gene expression signatures. Multiregion imaging features can identify and quantify changes in the microenvironment and ultimately reliably predict the cellular and molecular properties of tumors.

Moreover, we investigated whether a combined model integrating clinical, semantic features, and radiomics features would outperform the model using each feature alone. It could be found that, adding gender and deep white matter invasion to the radiomics features could not improve the performance of the model, and brought an insignificant performance decrease instead. This may be due to the fact that the clinical factors included were not comprehensive and that deep white matter invasion was a qualitative imaging factor that subjectively assessed by the radiologists, and could provide less diagnosis information than the deep mined radiomics features. The lower performance of the Clinical + Rad model compared to the ComRad model suggests potential overlap between some clinical features and the radiomics signature. Further experiments (Tables S23, S24) and detailed analysis confirmed that while the Radiomics signature demonstrates a robust predictive capability with a significant coefficient value (β = 9.714, *p* < 0.001), the clinical variables Gender and Deep_White_Matter_Invasion show relatively minimal impact (Gender: β = − 0.829, *p* = 0.569; Deep_White_Matter_Invasion: β = − 0.269, *p* = 0.843). The Kendall tau correlation results further suggest minimal overlap between these clinical variables and the Radiomics signature, with Gender and the Radiomics signature showing a tau of − 0.163 (*p* = 0.059) and Deep_White_Matter_Invasion and the Radiomics signature a tau of -0.098 (*p* = 0.257). These findings suggest that the addition of Gender and Deep_White_Matter_Invasion introduces redundancy without enhancing the model's predictive performance.These insightscould prompte us to refine our approach towards model construction. By elaborating on how future models might be optimized by focusing more on enhancing the quality and selection of radiomics features, potentially reducing reliance on less informative clinical variables. This nuanced understanding allows us to propose a more streamlined model that balances clinical relevance with predictive accuracy.

This study has several limitations. First, our study was a retrospective study of a multicenter cohort, and future prospective experiments using larger data sets from large-scale institutions should be conducted to develop more accurate and generalizable models. Second, we acquired four conventional MRI sequences, and these images are widely used in clinical practice. Studies have shown that MRI sequences such as DTI, DWI, and perfusion are promising to predict MGMT methylation^37,47,48^. These advanced MRI sequences can be incorporated into future studies to improve the predictive performance of the model. Third, we did not investigate the correlation of imaging features with other genetic molecular information, such as isocitrate dehydrogenase 1/2 (IDH-1/2), 1p/19q co-deletion, telomerase reverse transcriptase (TERT), Ki-67, etc. Additionally in image preprocessing, the Z-scoring method ensures that the data is scaled and evenly distributed before feature extraction, and enhances the consistency of our data analysis to a certain extent. However, current standardized approaches may not fully address all inter-device differences. Therefore, more advanced technologies, such as the ComBat algorithm, need to be introduced in the future to further correct possible batch effects between devices. We further analyzed misclassified images to understand model limitations. Misclassified cases often had large necrotic cores, significant and complex peritumoral tissues, which were particularly challenging. These findings suggest the need for improved preprocessing and additional features to capture these complexities. Detailed analysis is provided in the Supplementary file. Future work should refine preprocessing techniques and integrate advanced imaging features to enhance model performance and robustness.

## Conclusion

In conclusion, the multiregion radiomics model we established based on multi-sequence MRI can effectively predict the MGMT methylation status of GBM patients. The final comprehensive model that integrates multi-sequence and multi-habitat radiomics features demonstrates the best performance in discriminating the MGMT methylation status. This method shows potential in predicting molecular markers and can serve as a non-invasive and reliable preoperative assessment tool, marking a significant advancement in precision medicine that can guide individualized treatment decisions.

## Materials and methods

### Patients

This study was approved by the medical ethics committee of Zhongnan Hospital of Wuhan University (Approval number: 2021048), and all methods were performed in accordance with relevant guidelines and regulations. Due to the retrospective nature of this study and the CIOMS guidelines, a waiver of informed consent was obtained from the medical ethics committee of Zhongnan Hospital of Wuhan University. We consecutively and retrospectively collected patients with pathologically confirmed glioblastoma (2021 WHO classification of tumors of the central nervous System^49^) in our hospital from May 2017 to November 2021. Another part of the data from The Cancer Imaging Archive (TCIA, www.cancerimagingarchive.net) and The Cancer Genome Atlas (TCGA) public database. Finally, 100 patients from our hospital were included in this study and made up the training dataset, and 43 patients from the TCIA/TCGA dataset were assigned to the individual external validation dataset (test set 1). The details of patients recruitment was illustrated in Fig. S1. (Supplementary Method). We also collected 40 patients from Beijing Tiantan Hospital from April 2021 to May 2022 as test dataset 2, which were approved by the Ethics Committee of Beijing Tiantan Hospital (approval number: KY2022-078–04). All methods were performed in accordance with relevant guidelines and regulations, and informed consent was obtained from the Ethics Committee of Beijing Tiantan Hospital. Tables 1, 2 summarized the demographics and semantic imaging feature of the included patients in this study.

### Data flowchart

As shown in Fig. 1, the data processing flowchart of this study could be divided into two parts: Image analysis and machine learning modeling. The image analysis part included the following steps: image acquisition, preprocessing, gliomas region of interest (ROI) segmentation, and image features extraction. Each MRI sequence was first preprocessed to correct for field bias and resampled to a consistent resolution. ROIs were segmented using a 3D Unet model, and radiomics features were extracted from each ROI. The machine learning modeling part involved feature reduction using univariate logistic regression (uLR) and maximum relevance minimum redundancy (mRMR), followed by the construction of individual radiomics models using least absolute shrinkage and selection operator (LASSO). Finally, combination models were constructed and validated.

All 100 subjects from Zhongnan hospital were used as training set for the purpose of model construction, while the 43 subjects from TCIA and 40 subjects from Tiantan hospital were used for model external validation (Individual test set 1 and 2). Five-fold cross-validation was used in the model construction to explore the optimal model performance and to avoid the overfitting.

### MR image analysis

Preoperative MRI was performed with a 3.0 T scanner, including uMR 790 (United Imaging Healthcare), Magnetom Trio (Siemens Medical Solutions), Magnetom Prisma (Siemens Healthcare) or Ingenia CX (Philips Healthcare). For each patient, we collected the MR image data of four sequences: T1-weighted (T1), T1-weighted gadolinium contrast-enhanced (T1c), T2-weighted (T2) and T2-weighted FLAIR (T2f.). The detailed parameters of these sequences were shown in the supplementary Methods. T1, T1c and T2f. images are strictly registered with MATLAB software using T2 image as template. Then, all modals of images were preprocessed (details in Supplementary Methods) by simpleITK package, including N4 field bias correction, image resampling (1 mm*1 mm*5 mm) and z-score normalizing.

Next, an automatic VAT segmentation algorithm based on a 3D U-shape convolutional neural network (3D U-net, Fig. 2A,B) was proposed to automatically delimitate the gliomas ROI. The detailed of this model was shown in the Supplementary Method. In this study, three ROIs were defined. As shown in Fig. 2C, ROI1 (red) represented the “non-enhancing and edema” region (NE_EDEMA), which was defined as FLAIR hyperintense abnormality, excluding the contrast-enhancing and necrotic tumor region, ROI2 (green) the necrosis region (NEC), and ROI3 (blue) the contrast enhancement region (CE). The 3D U-net was first trained based on the 265 Brain tumor images with labels from the BraS 2015 database, and then used to segment the gliomas ROIs of the patients in this study. The segmented images were evaluated and then corrected by two radiologists using ITK-SNAP (www.itksnap.org) software in instances where tumor boundaries were challenging. Manual corrections were collaboratively performed by two junior radiologists, Li Lanqing and Wang Shouchao, under the oversight of senior radiologist Li Huan, who made final decisions in case of discrepancies. Neither of these radiologists was aware of the clinical and MGMT methylation information of the tumor.

Finally, Pyradiomics package (https://pyradiomics.readthedocs.io/en/latest/) was used to calculate the radiomics features of the three ROIs of the four image modals, and resulted in 1223 radiomics features (details in Supplementary Methods) for one ROI of each image modality in each patient. To assess the impact of the segmentation model on radiomics features and predictive performance, we performed a comparative analysis using both the 3D Unet model and manual segmentation by experienced radiologists. Detailed results of this analysis are provided in the Supplementary file.

### Machine learning modeling

First of all, we built prediction model for the specified ROI of the specified image modality respectively, which resulted in 12 individual models: T_i__R_j__score, where T_i_ represented the specified image modal (T1, T1c, T2 or T2f.) and R_j_ the specified ROI (ROI1, ROI2 or ROI3). During the T_i__R_j__score construction, we first use univariate Logstic Regression (uLR) to screen the features significantly correlated to the classification label. Then we applied the maximum relevance minimum redundancy (mRMR) algorithm to find the optimal feature subset through maximizing their relevance to the classification label while minimizing redundancy between features. Finally we used the least absolute shrinkage and selection operator (LASSO) methods to further select the radiomics features and to construct the individual model by imposing a penalty item to the modeling objective function.

Next, we built the combined models based on the constructed individual models. As shown in Table 3, four T_i_ models were constructed by the combination of three corresponding individual models constructed based on the Ti image modal (T_i__R1 + T_i__R2 + T_i__R3), and three R_j_ models were constructed by the combination of four corresponding individual models constructed based on the Rj ROI (T1_R_j_ + T1c_R_j_ + T2_R_j_ + T2f_R_j_). All 12 individual models were combined to construct the ComRad model. Clinical characteristics and semantic features were also analyzed to establish the Clinical model, which was then incorporated into the Clinical + Rad model construction. The specified combination method used for different combination models in this study was also shown in Table 3.

At last, all constructed models were validated and compared. Their prediction accuracy were evaluated using the receiver operator characteristic (ROC) and the related four metrics: Area under the curve (AUC), accuracy, sensitivity and specificity. Delong test was used to validate whether the performance difference between the models was significant. In addition, the clinical usability of the models were assessed using the model net benefit showed in decision curve.

### Statistics

In this study, the 3D Unet model was implemented using Pytorch 1.13.1 and python 3.7.6. Other statistical analysis and machine learning modeling was performed using R 4.3.0. “mRMRe” and “glmnet” packages were used to realize the mRMR and LASSO method respectively, and the “pROC” and “dcurves” package was used to draw the ROC curve and DCA curve respectively, and then compute relevant indicators for the model validation and comparison.

### Supplementary Information

Supplementary Information.

## Data Availability

The datasets generated during and/or analysed during the current study are available from the corresponding author on reasonable request.
