# Supplementary material for: Preoperative prediction of MGMT promoter methylation in glioblastoma based on multiregional and multi-sequence MRI radiomics analysis
Source: Sci Rep. 2024 Jul 11;14:16031. doi: 10.1038/s41598-024-66653-2 (PMC11239670; doi:10.1038/s41598-024-66653-2)
Supplement: Supplementary file 1 — Supplementary Information. [file 41598_2024_66653_MOESM1_ESM.docx]

**Supplementary Methods and Results**

**Title Page**

**Preoperative prediction of MGMT promoter methylation in glioblastoma based on multiregional and multi-sequence MRI radiomics analysis**

**Lanqing Li1#,** **Feng Xiao1#, Shouchao Wang2, Shengyu Kuang1, Zhiqiang Li3, Yahua Zhong4, Dan Xu5, Yuxiang Cai6, Sirui Li1, Jun Chen7, Yaou Liu8, Junjie Li8, Huan Li1*, and Haibo Xu1***

**Patients Enrollment**

As the inclusion criteria for patient shown in sFig. 1, we searched the imaging data of all hospitalized patients in the PACS system from May 2018 to November 2021, and found a total of 199 institutional patients with pathologically confirmed glioblastoma, of which 160 patients had available MGMT methylation status. Then we checked the image sequence integrity and the image quality of these patients and resulted in 100 subjects, which were used for the model construction. Similarly as shown in sFig. 1, we found 43 subjects in the TCIA database, and used as individual external test set 1. We also collected 40 patients from Beijing Tiantan Hospital during the period from April 2021 to May 2022 as test set 2.

**MR Images Acquisition**

Preoperative MRI was performed with a 3.0T scanner (UIH, Siemens or Philips). Four image sequences (T1, T1c, T2 and T2f) were acquired for each patient, and the details of parameters were as follow:

UIH scanner：T1WI（TR =7.1ms；TE =3.1ms；FOV =256×256mm2；matrix: 256×256；Slice thickness= 1mm） ;T2WI（TR=5100ms；TE=133ms；FOV = 230×200 mm2；matrix:384×326；Slice thickness= 5mm） ;T2FLAIR（TR=6000ms；TE=400ms；FOV = 256×256 mm2；matrix:256×256；Slice thickness= 1mm） ;CE-T1WI （TR=7.1ms；TE=3.1ms；FOV =256×256 mm2；matrix：256×256；Slice thickness = 1mm；CE-T1WI was obtained after the injection of 0.1 mmol/kg of Gd-DTPA）.

Siemens scanner：T1WI（TR =2000-2300ms；TE =2.3-13ms；FOV = 250×250 mm2；matrix:256×256；Slice thickness=1mm） ;T2WI（TR=4000-5000ms；TE=89-103ms；FOV = 220×220 mm2；matrix:512×410；Slice thickness=5mm） ;T2-FLAIR（TR=7000-8000ms；TE=80-110ms；FOV =250×250 mm2；matrix: 256×256；Slice thickness= 4-6mm） ;CE-T1WI （TR=1900-2000ms；TE=2.3-4.4ms；FOV = 240×225 mm2；matrix：256×240；Slice thickness =1mm；CE-T1WI was obtained after the injection of 0.1 mmol/kg of Gd-DTPA）.

Philips scanner：T1WI（TR = 6-6.6ms；TE = 2-3.1ms；FA = 8°；Slice thickness= 1-5mm） ;T2WI（TR= 2800-4600ms；TE= 100-135ms；FA = 90°；Slice thickness= 5mm） ;T2FLAIR（TR= 4800ms；TE= 256ms；FA = 90°；TI = 1650ms；Slice thickness= 5mm） ;CE-T1WI （TR= 8-2000ms；TE= 2-20ms；FA = 8-90°；TI = 800ms；Slice thickness = 1-5mm）.

**Image Preprocess**

Considering the variability in images from different vendors, we applied several preprocessing steps to ensure consistency. These included:

1. N4 field bias correction: This was performed to correct for intensity non-uniformities in the MR images.

2. Image resampling: All images were resampled to a common resolution of 1mm×1mm×5mm using bilinear interpolation method to ensure spatial consistency across different datasets.

3. Z-score normalization: This was used to standardize the intensity values across different scanners, ensuring that the images have a mean of 0 and a standard deviation of 1. This step is crucial for reducing the variability introduced by different MRI machines and settings.

These preprocessing steps were carefully chosen to harmonize the imaging data, thereby improving the reliability and reproducibility of the radiomics analysis.

**Radiomics Features**

Radiomics features were extracted based on different ROIs (NE_EDEMA, NEC and CE area) of different MR images (T1c, T1, T2 and T2f) using pyradiomics codes (https://pyradiomics.readthedocs.io/en/latest/). Three categories of features were calculated in this study. The first category was the original radiomics features, which contained the classes of First order statistics, Shape and texture features while the texture features contained Gray level co-occurrence matrix(GLCM), Gray level Run-length matrix(GLRLM), Gray level size zone matrix(GLSZM), Neighboring Gray Tone Difference Matrix(NGTDM) and [Gray Level Dependence Matrix](https://www.abbreviationfinder.org/cn/acronyms/gldm_gray-level-dependence-matrix.html)(GLDM)**.** These original radiomics features were calculated based on the original images and VOIs. Besides, we also extracted two categories of high order features: first processed the original images using different filters or mathematical transformations, then extracted their first order statistics, shape and texture features using pyradiomics method, which was similar to the calculation of original features. Laplacian of Gaussian (LoG) and Wavelet filters with different inner parameters were adopted in the generation of high order. Finally, a total of 1223 radiomics features (Original: 107; LoG: 372; Wavelet: 744) were extracted based on each ROI on each MR modal for each patient.

The description and calculation details for all radiomics features could be found in the online pyradiomics docs. (<https://pyradiomics.readthedocs.io/en/latest/features.html>).

**3D U-shape CNN Gliomas segmentation model**

In this study, we have constructed an automatic VAT segmentation algorithm based on a 3D U-shape convolutional neural network (Fig.2A). The topology of the whole network consists of the encoding and decoding parts (Fig.2B). There are five stages in the encoding and decoding subnetwork, indicating that five-level scales of feature maps were formulated for automatic feature extraction. In the encoding part, each stage consists of two convolutional layers with 3x3x3 kernel and a max pooling layer with 2x2x2 kernel and 2x2x2 stride to compress spatial information; In the decoding part, each stage consists of two convolutional layers with 3x3x3 kernel and a transpose convolutional layer with 3x3x3 kernel and 2x2x2 stride for VAT feature reconstruction.

Before being fed into the neural network, spatial and intensity normalization was used to eliminate heterogeneity of different machines and parameters of the scanners. For spatial normalization, the images firstly were cropped to filter the void area, and then resample into the spacing of 1.52*1.52*2.74, final cropped randomly into 112*160*128 for data augmentation. For intensity normalization, after filtering the void area, we collect and clip the image into their 0.05% and 99.5% intensity values and use z-score normalization.

**Evaluation of the impact for different Segmentation Methods**

To assess the impact of the segmentation model, we performed a comparative analysis using both the 3D Unet model and manual segmentation by the experienced radiologists. Radiomics features were extracted from the segmented ROIs for both methods using the PyRadiomics package. Additionally, we evaluated the influence of these segmentation methods on the predictive performance of our radiomics model.

The analysis showed high correlation coefficients for most radiomics features between the 3D Unet and manual segmentation methods (correlation coefficient: >0.75), with minor differences observed in certain texture features such as entropy (correlation coefficient: 0.71) and uniformity (correlation coefficient: 0.68). For predictive performance, the AUC for the 3D Unet segmentation was 0.839 (95% CI: 0.709-0.963) on test set 1 and 0.739 (95% CI: 0.581-0.897) on test set 2. The AUC for manual segmentation was 0.831 (95% CI: 0.701-0.961) on test set 1 and 0.732 (95% CI: 0.574-0.890) on test set 2.

In conclusion, while the majority of radiomics features are consistent across different segmentation methods, some texture features can vary. Both segmentation methods provide reliable results for radiomics feature extraction and predictive performance, but caution should be exercised when interpreting specific texture features. The automated 3D Unet segmentation is a valid and robust approach for this study.

**Misclassified Image Analysis**

To further understand the limitations of our models, we conducted an analysis of the misclassified images. Several common characteristics were identified among the misclassified cases:

(1) Extensive Necrotic Regions: Images with large necrotic areas often exhibited irregular enhancement patterns, which could lead to incorrect classification.

(2) Significant Surrounding Tissues Presence: The presence of nearby structures around the tumor such as blood vessels, skull, and cerebrospinal fluid (CSF) can complicate the segmentation and affect feature extraction.

To illustrate these common characteristics, three Case Examples were listed:

Case 1 (Figure S7): A case with a large necrotic core was misclassified, likely due to the atypical appearance of the tumor.

Case 2 (Figure S8): A case with significant peritumoral edema near the skull resulted in poor feature extraction, leading to misclassification.

Case 3 (Figure S9): A case with both extensive necrosis and proximity to cerebrospinal fluid was misclassified, highlighting the challenges in these complex scenarios.

**Code Availability on GitHub**

We have uploaded the complete sets of scripts used for image processing and machine learning modeling related to our study. These are now publicly accessible in two separate GitHub repositories:

(1) For image processing and machine learning modeling components, please visit: <https://github>.com/seiya0731/GBM-MGMT_Prediction

(2) For the automatic segmentation of gliomas using the 3D U-Net model, please access: <https://github>.com/CharelBIT/nnUNet-modify

**Supplementary Figures**


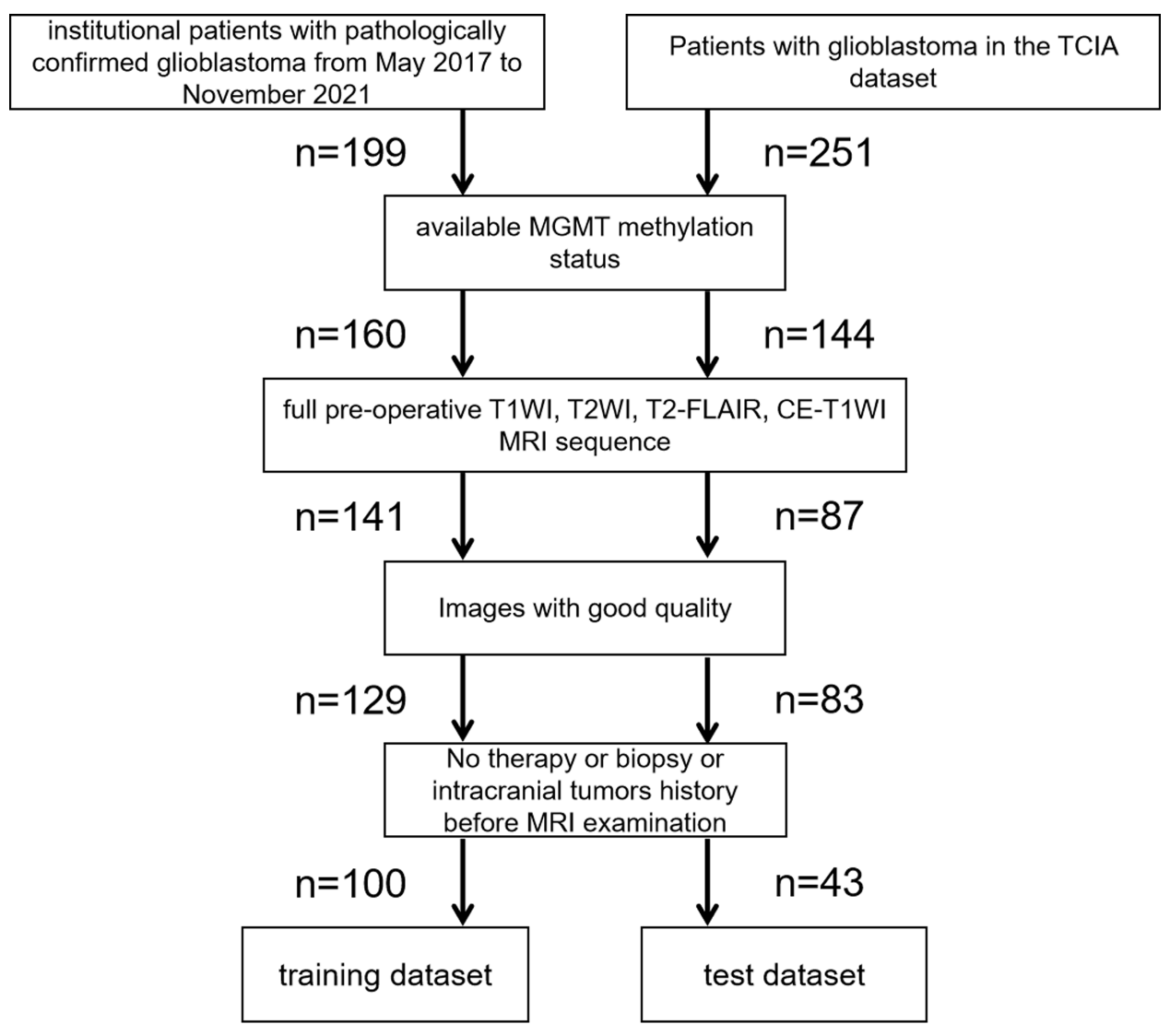


**Figure S1 Flowchart of patient selection of this study.** TCIA: The Cancer Imaging Archive.

**
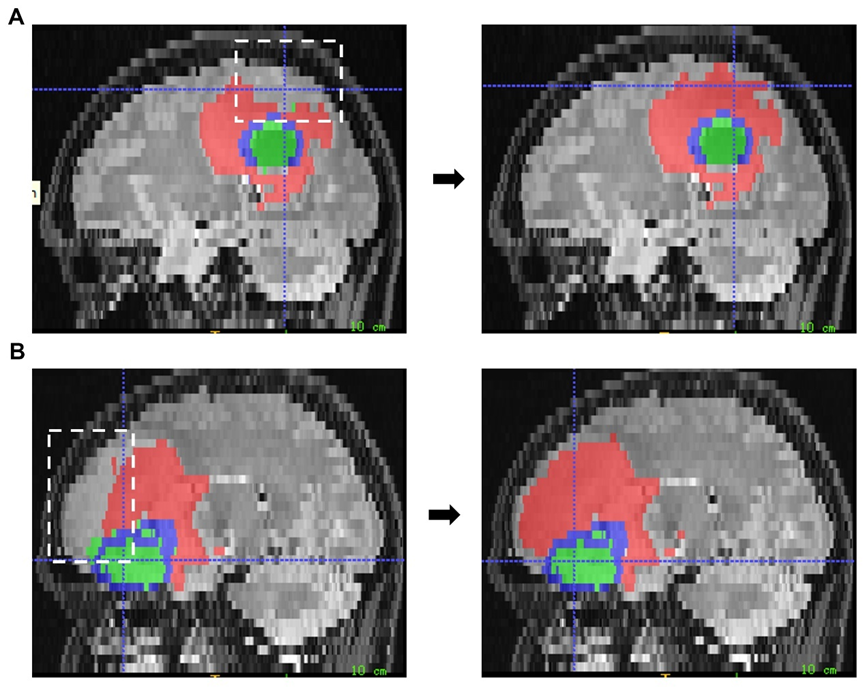
Figure S2 Examples for the manual correction after the 3D U-Net model automatical segmentation.** Two typical automatically segmented examples, in which edema areas were not fully recognized and need to be corrected manually.

**
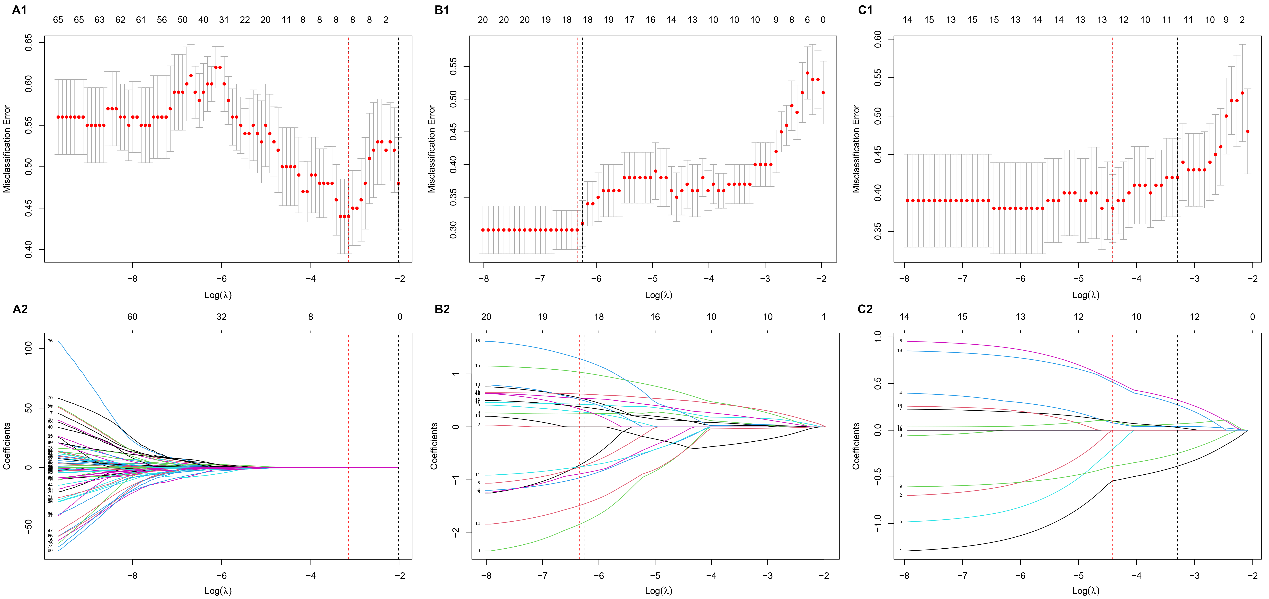
**

**Figure S3 Feature selection and modeling using LASSO method based on the three ROIs in the T1c images.** From left to right, the modeling process of 3 different models based on three different ROIs (NE_EDEMA, NEC and CE area) were shown. From top to bottom, (A) showed the determination of the key parameter (penalty coefficient: λ) in the LASSO model using 10-fold cross-validation. Two rules resulted in two λ values (λ_min_: when the misclassification error reached the minimum and λ_1se_: the value lead to the minimum number of features, as well as the misclassification error restricted within one times standard error from the minimum) and two vertical dashed lines at their position were drawn. Λ_min_ was adopted in the feature selection of LASSO in this study; (B) showed the feature coefficients profiles as the λ value changes. According to the 10-fold cross-validation in (A), the features with non-zero coefficients were selected at the position of λ_min_ for further modeling. (A1-A2) λ_min_=0.0428 with log(λ_min_)=-3.1511 was determined, in which 8 features with nonzero coefficients were finally selected for the T1c_R1_Score model construction. (B1-B2) λ_min_=0.0018 with log(λ_min_)=--6.3408 was determined, in which 18 features with nonzero coefficients were finally selected for the T1c_R2_Score model construction. (C1-C2) λ_min_=0.0121 with log(λ_min_)=--4.4108 was determined, in which 10 features with nonzero coefficients were finally selected for the T1c_R3_Score model construction.

**
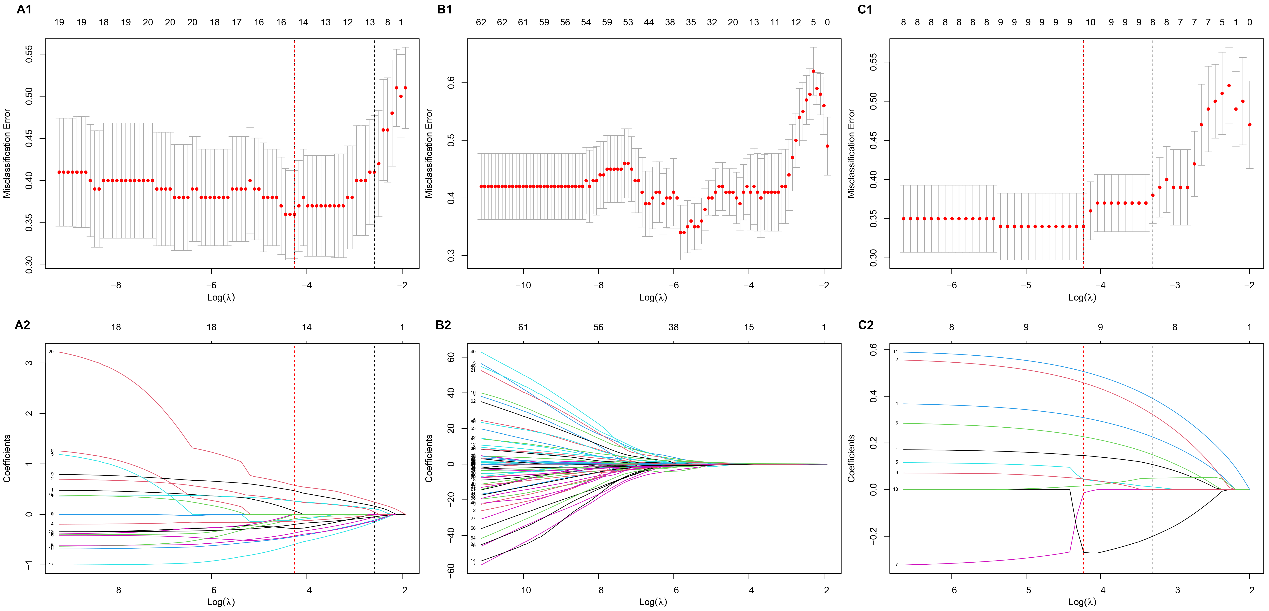
**

**Figure S4 Feature selection and modeling using LASSO method based on the three ROIs in the T1 images.** From left to right, the modeling process of 3 different models based on three different ROIs (NE_EDEMA, NEC and CE area) were shown. From top to bottom, (A) showed the determination of the key parameter (penalty coefficient: λ) in the LASSO model using 10-fold cross-validation. Two rules resulted in two λ values (λ_min_: when the misclassification error reached the minimum and λ_1se_: the value lead to the minimum number of features, as well as the misclassification error restricted within one times standard error from the minimum) and two vertical dashed lines at their position were drawn. Λ_min_ was adopted in the feature selection of LASSO in this study; (B) showed the feature coefficients profiles as the λ value changes. According to the 10-fold cross-validation in (A), the features with non-zero coefficients were selected at the position of λ_min_ for further modeling. (A1-A2) λ_min_=0.0141 with log(λ_min_)=-4.2595 was determined, in which 14 features with nonzero coefficients were finally selected for the T1_R1_Score model construction. (B1-B2) λ_min_=0.0033 with log(λ_min_)=-5.7249 was determined, in which 38 features with nonzero coefficients were finally selected for the T1_R2_Score model construction. (C1-C2) λ_min_=0.0146 with log(λ_min_)=-4.2275 was determined, in which 10 features with nonzero coefficients were finally selected for the T1_R3_Score model construction.

**
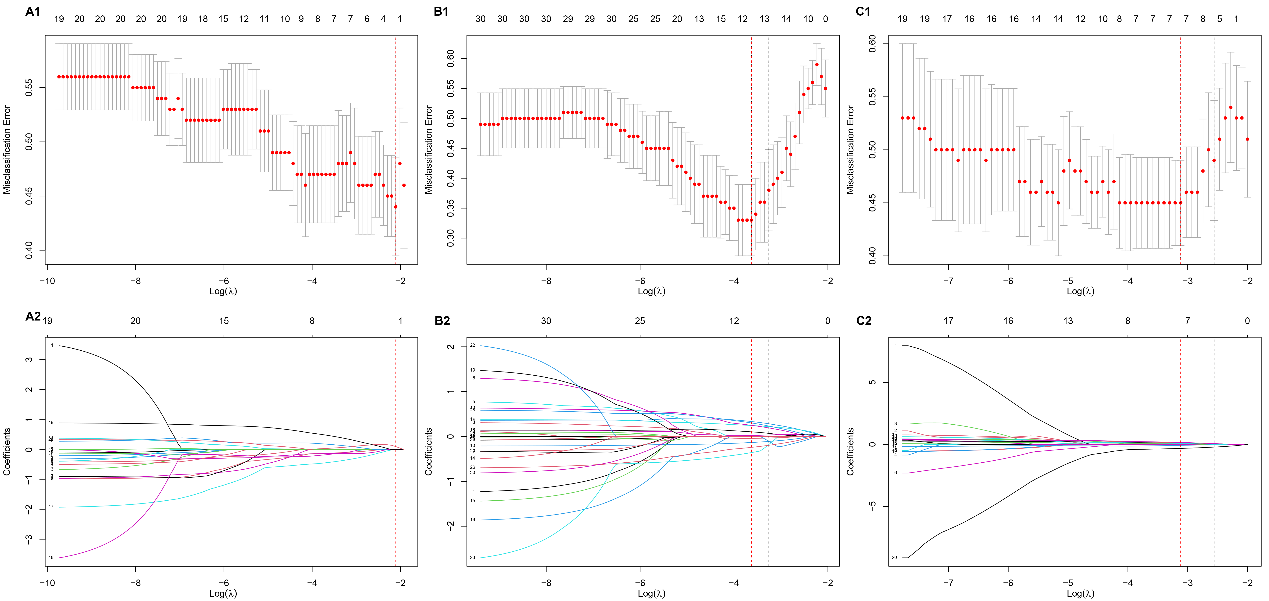
**

**Figure S5 Feature selection and modeling using LASSO method based on the three ROIs in the T2 images.** From left to right, the modeling process of 3 different models based on three different ROIs (NE_EDEMA, NEC and CE area) were shown. From top to bottom, (A) showed the determination of the key parameter (penalty coefficient: λ) in the LASSO model using 10-fold cross-validation. Two rules resulted in two λ values (λ_min_: when the misclassification error reached the minimum and λ_1se_: the value lead to the minimum number of features, as well as the misclassification error restricted within one times standard error from the minimum) and two vertical dashed lines at their position were drawn. Λ_min_ was adopted in the feature selection of LASSO in this study; (B) showed the feature coefficients profiles as the λ value changes. According to the 10-fold cross-validation in (A), the features with non-zero coefficients were selected at the position of λ_min_ for further modeling. (A1-A2) λ_min_=0.1217 with log(λ_min_)=-2.1059 was determined, in which 1 features with nonzero coefficients were finally selected for the T2_R1_Score model construction. (B1-B2) λ_min_=0.0265 with log(λ_min_)=-3.6290 was determined, in which 12 features with nonzero coefficients were finally selected for the T2_R2_Score model construction. (C1-C2) λ_min_=0.0443 with log(λ_min_)=-3.1167 was determined, in which 7 features with nonzero coefficients were finally selected for the T2_R3_Score model construction.

**
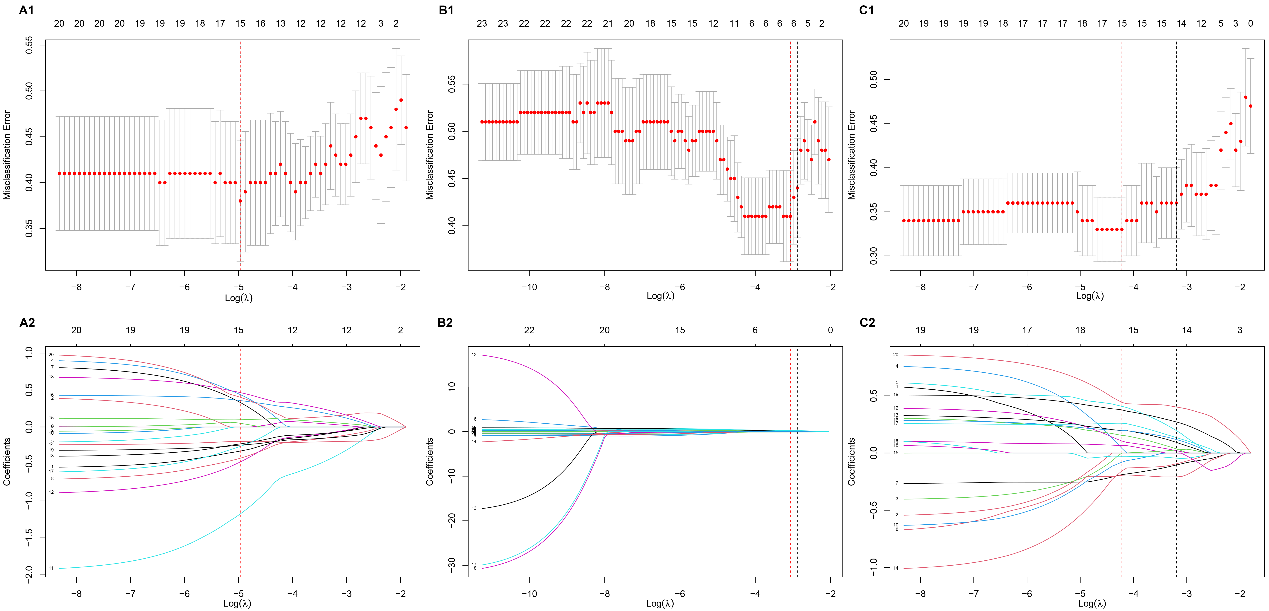
**

**Figure S6 Feature selection and modeling using LASSO method based on the three ROIs in the T2f images.** From left to right, the modeling process of 3 different models based on three different ROIs (NE_EDEMA, NEC and CE area) were shown. From top to bottom, (A) showed the determination of the key parameter (penalty coefficient: λ) in the LASSO model using 10-fold cross-validation. Two rules resulted in two λ values (λ_min_: when the misclassification error reached the minimum and λ_1se_: the value lead to the minimum number of features, as well as the misclassification error restricted within one times standard error from the minimum) and two vertical dashed lines at their position were drawn. Λ_min_ was adopted in the feature selection of LASSO in this study; (B) showed the feature coefficients profiles as the λ value changes. According to the 10-fold cross-validation in (A), the features with non-zero coefficients were selected at the position of λ_min_ for further modeling. (A1-A2) λ_min_=0.0070 with log(λ_min_)=-4.9680 was determined, in which 15 features with nonzero coefficients were finally selected for the T1c_R1_Score model construction. (B1-B2) λ_min_=0.0465 with log(λ_min_)=-3.0687 was determined, in which 6 features with nonzero coefficients were finally selected for the T1c_R2_Score model construction. (C1-C2) λ_min_=0.0147 with log(λ_min_)=-4.2180 was determined, in which 15 features with nonzero coefficients were finally selected for the T1c_R3_Score model construction.


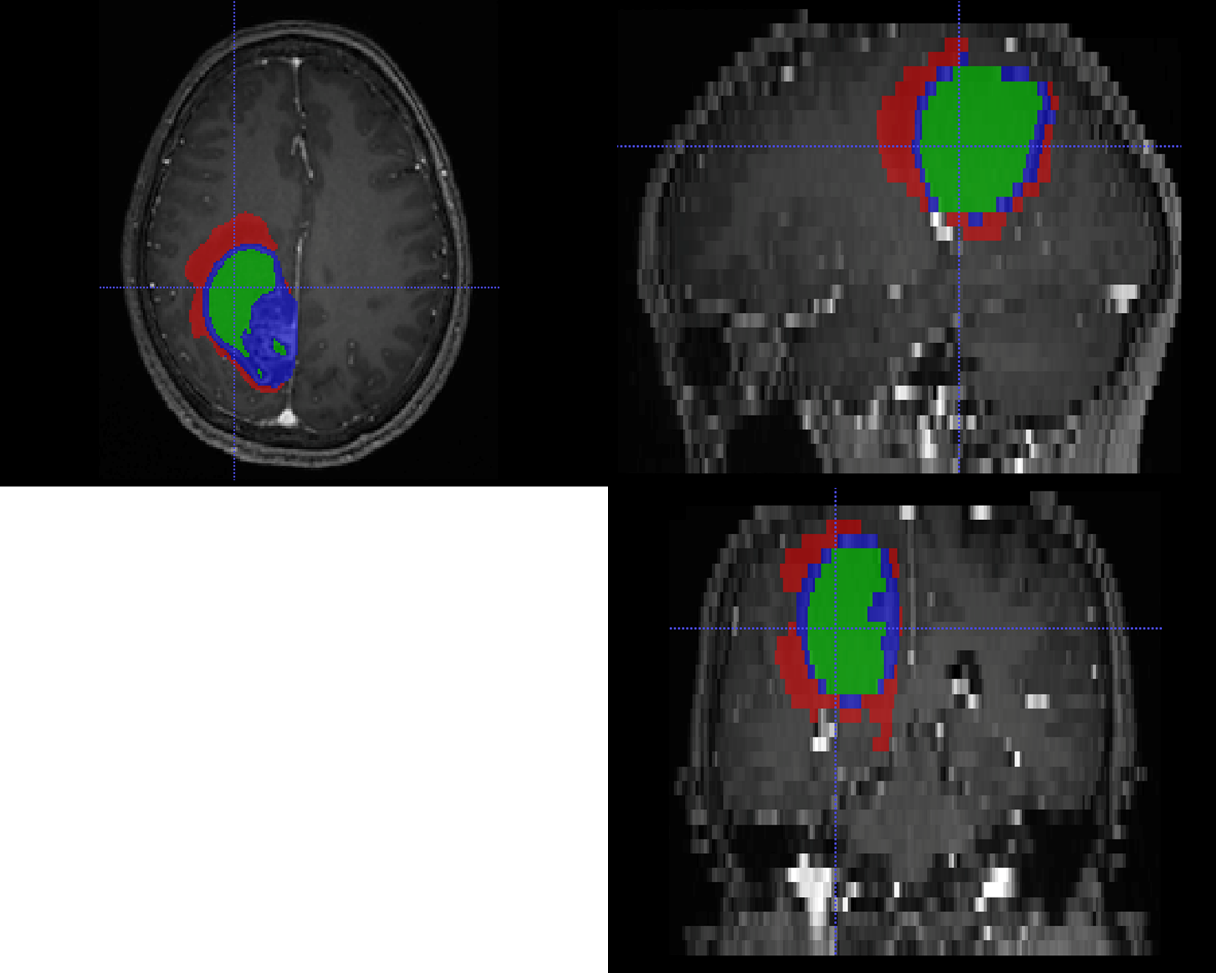


**Figure S7 Example of a case with a large necrotic core and irregular enhancement pattern.**


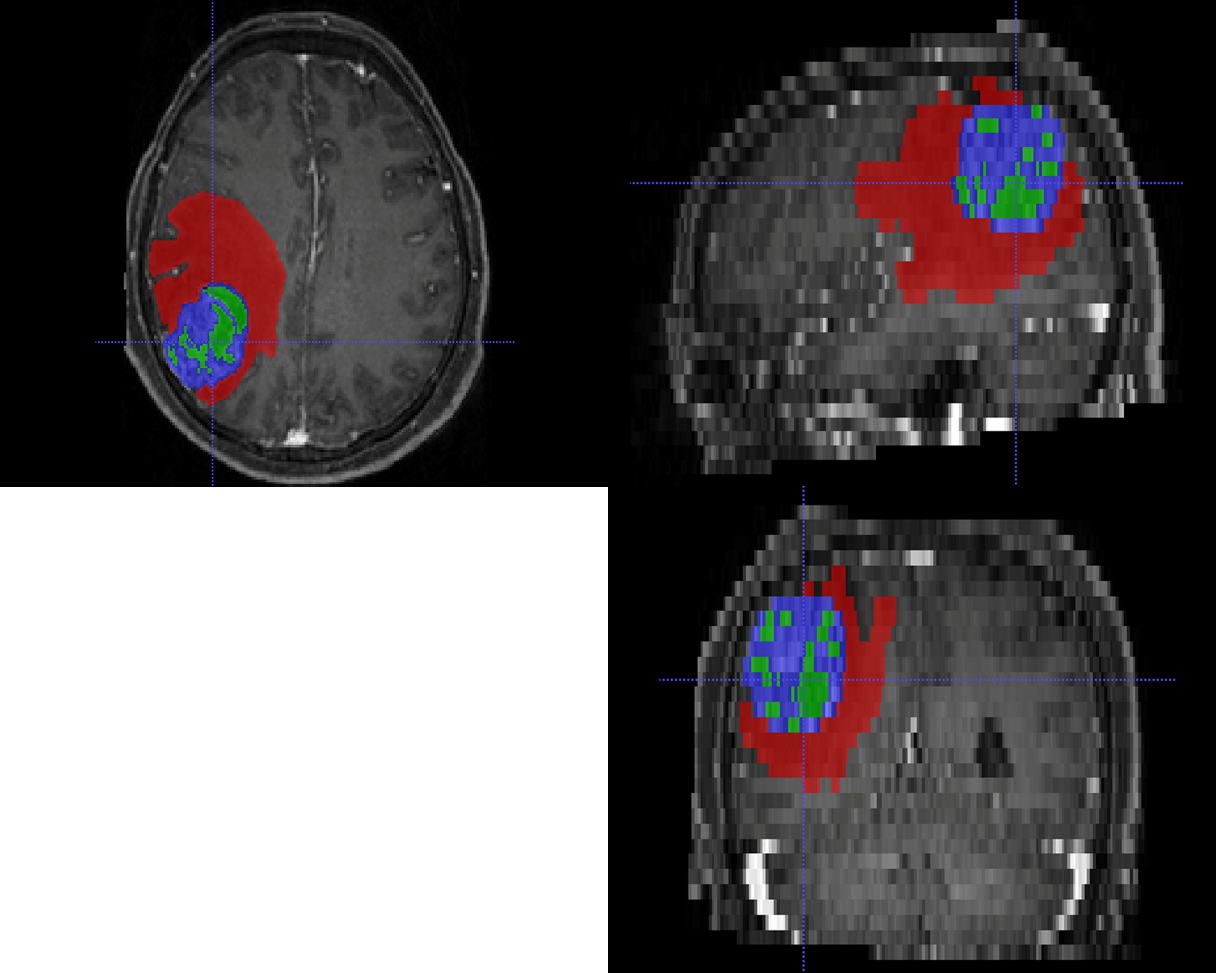


**Figure S8 Example of a case with the tumor near the skull.**


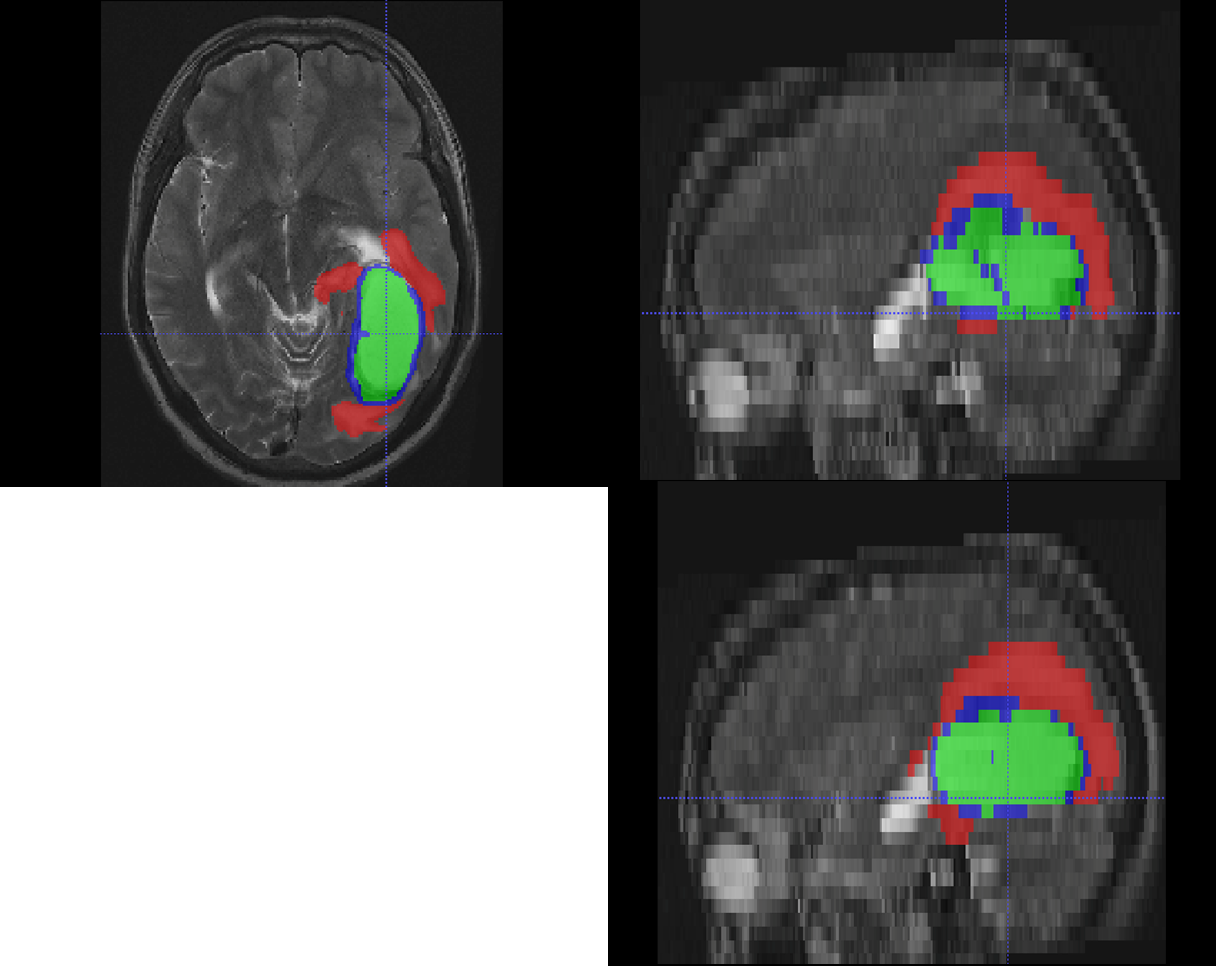


**Figure S9 Example of a case with extensive necrosis and proximity to cerebrospinal fluid.**

**Supplementary Tables**

**Table S1 T1c_R1 radiomics features selected for T1c_R1_Score model construction using LASSO method.**

| index | Feature Name | Weighted. |
| --- | --- | --- |
| 0 | (Intercept) | -0.118996616 |
| 1 | original_glszm_HighGrayLevelZoneEmphasis_t1c_1 | 0.175476492 |
| 2 | original_glszm_LowGrayLevelZoneEmphasis_t1c_1 | -3.22E-15 |
| 3 | original_glszm_SmallAreaLowGrayLevelEmphasis_t1c_1 | -0.093666018 |
| 4 | log.sigma.4.0.mm.3D_glcm_ClusterShade_t1c_1 | 0.120053091 |
| 5 | log.sigma.5.0.mm.3D_glrlm_GrayLevelNonUniformityNormalized_t1c_1 | 0.180292074 |
| 6 | wavelet.HLH_firstorder_Median_t1c_1 | 0.124827508 |
| 7 | wavelet.HHL_glszm_ZonePercentage_t1c_1 | 0.173469579 |
| 8 | wavelet.LLL_firstorder_Skewness_t1c_1 | -0.122986922 |

**Table S2 T1c_R2 radiomics features selected for T1c_R2_Score model construction using LASSO method.**

| index | Feature Name | Weighted. |
| --- | --- | --- |
| 0 | (Intercept) | -0.175364171 |
| 1 | wavelet.HHH_firstorder_TotalEnergy_t1c_2 | -0.720334345 |
| 2 | log.sigma.2.0.mm.3D_gldm_DependenceEntropy_t1c_2 | -1.838087107 |
| 3 | log.sigma.2.0.mm.3D_glszm_SizeZoneNonUniformity_t1c_2 | -0.961545143 |
| 4 | log.sigma.2.0.mm.3D_gldm_DependenceVariance_t1c_2 | 0.284599016 |
| 5 | log.sigma.5.0.mm.3D_glrlm_GrayLevelNonUniformityNormalized_t1c_2 | -0.88722649 |
| 6 | wavelet.HLL_firstorder_Energy_t1c_2 | 0.54616715 |
| 7 | original_glszm_LargeAreaEmphasis_t1c_2 | -0.757523844 |
| 8 | wavelet.HLH_gldm_DependenceVariance_t1c_2 | 0.247346927 |
| 9 | log.sigma.3.0.mm.3D_ngtdm_Busyness_t1c_2 | 0.517280073 |
| 10 | log.sigma.5.0.mm.3D_glcm_ClusterProminence_t1c_2 | -0.755004857 |
| 11 | wavelet.LHH_firstorder_TotalEnergy_t1c_2 | 0.338997224 |
| 12 | log.sigma.5.0.mm.3D_glszm_SizeZoneNonUniformity_t1c_2 | 0.381494667 |
| 13 | log.sigma.2.0.mm.3D_gldm_DependenceNonUniformityNormalized_t1c_2 | -1.482503988 |
| 14 | log.sigma.3.0.mm.3D_glcm_Correlation_t1c_2 | 1.032090918 |
| 15 | original_glszm_LargeAreaHighGrayLevelEmphasis_t1c_2 | 1.287273837 |
| 16 | wavelet.LLH_glszm_SizeZoneNonUniformity_t1c_2 | 0.427234349 |
| 17 | log.sigma.5.0.mm.3D_glcm_MCC_t1c_2 | 0.542132445 |
| 18 | wavelet.LLH_glszm_GrayLevelVariance_t1c_2 | 0.621365719 |
|  |  |  |

**Table S3 T1c_R3 radiomics features selected for T1c_R3_Score model construction using LASSO method.**

| index | Feature Name | Weighted. |
| --- | --- | --- |
| 0 | (Intercept) | -0.103642578 |
| 1 | log.sigma.3.0.mm.3D_glcm_Imc1_t1c_3 | -0.542766775 |
| 2 | log.sigma.5.0.mm.3D_glszm_SizeZoneNonUniformity_t1c_3 | 0.077876524 |
| 3 | wavelet.LLH_glszm_SizeZoneNonUniformity_t1c_3 | -0.200570039 |
| 4 | wavelet.LLH_glszm_SizeZoneNonUniformityNormalized_t1c_3 | 0.54079696 |
| 5 | wavelet.LHH_firstorder_Energy_t1c_3 | 0.09785852 |
| 6 | wavelet.LHH_glcm_Contrast_t1c_3 | -0.386571871 |
| 7 | wavelet.LHH_glcm_Id_t1c_3 | 3.65E-13 |
| 8 | wavelet.LHH_glcm_Idm_t1c_3 | 4.98E-16 |
| 9 | wavelet.HLH_glszm_SizeZoneNonUniformity_t1c_3 | 0.096387072 |
| 10 | wavelet.HHH_glszm_SizeZoneNonUniformity_t1c_3 | 0.512372442 |

**Table S4 T1_R1 radiomics features selected for T1_R1_Score model construction using LASSO method.**

| index | Feature Name | Weighted. |
| --- | --- | --- |
| 0 | (Intercept) | -0.229893652 |
| 1 | wavelet.HLL_gldm_LargeDependenceLowGrayLevelEmphasis_t1_1 | 0.07000185 |
| 2 | log.sigma.5.0.mm.3D_firstorder_Minimum_t1_1 | 0.26207211 |
| 3 | log.sigma.5.0.mm.3D_firstorder_90Percentile_t1_1 | -0.084333692 |
| 4 | wavelet.HHH_glcm_Autocorrelation_t1_1 | -0.111027953 |
| 5 | wavelet.LHL_glcm_Imc2_t1_1 | -0.367501454 |
| 6 | log.sigma.5.0.mm.3D_firstorder_Variance_t1_1 | -0.280163712 |
| 7 | log.sigma.3.0.mm.3D_firstorder_Minimum_t1_1 | 0.261795139 |
| 8 | wavelet.HHH_glszm_SizeZoneNonUniformityNormalized_t1_1 | -0.198402865 |
| 9 | log.sigma.5.0.mm.3D_glcm_ClusterProminence_t1_1 | -0.135384292 |
| 10 | wavelet.HHL_firstorder_Mean_t1_1 | -0.441718123 |
| 11 | wavelet.LLH_glszm_SmallAreaHighGrayLevelEmphasis_t1_1 | -0.590756665 |
| 12 | log.sigma.5.0.mm.3D_glcm_Autocorrelation_t1_1 | -0.426088519 |
| 13 | wavelet.LHL_glcm_DifferenceVariance_t1_1 | 0.469275513 |
| 14 | wavelet.HHH_gldm_LowGrayLevelEmphasis_t1_1 | 0.575390591 |
|  |  |  |

**Table S5 T1_R2 radiomics features selected for T1_R2_Score model construction using LASSO method.**

| index | Feature Name | Weighted. |
| --- | --- | --- |
| 0 | (Intercept) | -0.37217 |
| 1 | log.sigma.2.0.mm.3D_glcm_SumEntropy_t1_2 | -1.40583 |
| 2 | log.sigma.2.0.mm.3D_glcm_SumSquares_t1_2 | 2.505796 |
| 3 | log.sigma.2.0.mm.3D_ngtdm_Complexity_t1_2 | -2.66064 |
| 4 | log.sigma.2.0.mm.3D_gldm_DependenceEntropy_t1_2 | -0.88273 |
| 5 | log.sigma.2.0.mm.3D_gldm_DependenceNonUniformityNormalized_t1_2 | -1.02106 |
| 6 | log.sigma.3.0.mm.3D_glcm_Imc2_t1_2 | 0.826406 |
| 7 | log.sigma.3.0.mm.3D_glcm_SumSquares_t1_2 | -0.06157 |
| 8 | log.sigma.3.0.mm.3D_ngtdm_Contrast_t1_2 | 2.33252 |
| 9 | log.sigma.3.0.mm.3D_gldm_DependenceEntropy_t1_2 | -0.2539 |
| 10 | log.sigma.4.0.mm.3D_glcm_ClusterTendency_t1_2 | 1.076645 |
| 11 | log.sigma.4.0.mm.3D_glcm_JointEntropy_t1_2 | -3.50922 |
| 12 | log.sigma.4.0.mm.3D_glcm_SumEntropy_t1_2 | -0.00608 |
| 13 | log.sigma.4.0.mm.3D_glrlm_RunLengthNonUniformity_t1_2 | -0.43229 |
| 14 | log.sigma.4.0.mm.3D_ngtdm_Busyness_t1_2 | 0.385087 |
| 15 | log.sigma.5.0.mm.3D_glcm_Contrast_t1_2 | 1.731079 |
| 16 | log.sigma.5.0.mm.3D_glcm_DifferenceAverage_t1_2 | 1.27E-16 |
| 17 | log.sigma.5.0.mm.3D_glcm_InverseVariance_t1_2 | 6.48E-17 |
| 18 | log.sigma.5.0.mm.3D_glcm_MaximumProbability_t1_2 | 1.032176 |
| 19 | log.sigma.5.0.mm.3D_glrlm_GrayLevelNonUniformityNormalized_t1_2 | 0.008212 |
| 20 | log.sigma.5.0.mm.3D_glrlm_GrayLevelVariance_t1_2 | -0.01066 |
| 21 | log.sigma.5.0.mm.3D_glszm_SizeZoneNonUniformity_t1_2 | 0.171959 |
| 22 | log.sigma.5.0.mm.3D_ngtdm_Busyness_t1_2 | 0.003132 |
| 23 | log.sigma.5.0.mm.3D_ngtdm_Strength_t1_2 | -0.68733 |
| 24 | log.sigma.5.0.mm.3D_gldm_GrayLevelVariance_t1_2 | 0.290048 |
| 25 | wavelet.LLH_glcm_ClusterTendency_t1_2 | -0.32658 |
| 26 | wavelet.LLH_glcm_DifferenceVariance_t1_2 | 1.181866 |
| 27 | wavelet.LLH_glcm_Imc1_t1_2 | -1.31469 |
| 28 | wavelet.LLH_glcm_Imc2_t1_2 | -0.94902 |
| 29 | wavelet.LLH_glcm_JointEntropy_t1_2 | -0.03115 |
| 30 | wavelet.LLH_glcm_MaximumProbability_t1_2 | -0.67478 |
| 31 | wavelet.LLH_glszm_SizeZoneNonUniformity_t1_2 | 0.1905 |
| 32 | wavelet.LHH_glcm_MCC_t1_2 | -0.07102 |
| 33 | wavelet.HLL_glcm_SumSquares_t1_2 | 0.091642 |
| 34 | wavelet.HLH_glszm_GrayLevelNonUniformityNormalized_t1_2 | -0.80731 |
| 35 | wavelet.HHL_firstorder_Mean_t1_2 | -0.16117 |
| 36 | wavelet.HHH_firstorder_Entropy_t1_2 | 1.550314 |
| 37 | wavelet.HHH_glcm_MaximumProbability_t1_2 | -0.79146 |
| 38 | wavelet.HHH_glcm_SumSquares_t1_2 | -0.81755 |

**Table S6 T1_R3 radiomics features selected for T1_R3_Score model construction using LASSO method.**

| index | Feature Name | Weighted. |
| --- | --- | --- |
| 0 | (Intercept) | -0.11109 |
| 1 | log.sigma.2.0.mm.3D_glszm_SizeZoneNonUniformity_t1_3 | 0.146026 |
| 2 | log.sigma.3.0.mm.3D_glszm_SizeZoneNonUniformityNormalized_t1_3 | 0.457738 |
| 3 | log.sigma.4.0.mm.3D_glszm_SmallAreaHighGrayLevelEmphasis_t1_3 | 0.226736 |
| 4 | log.sigma.5.0.mm.3D_ngtdm_Busyness_t1_3 | 0.308495 |
| 5 | wavelet.LLH_glcm_DifferenceVariance_t1_3 | 0.043244 |
| 6 | wavelet.LLH_glcm_Imc2_t1_3 | -0.01397 |
| 7 | wavelet.LLH_glcm_MCC_t1_3 | -0.26891 |
| 8 | wavelet.LLH_glszm_GrayLevelNonUniformity_t1_3 | 0.045651 |
| 9 | wavelet.LLH_glszm_SizeZoneNonUniformity_t1_3 | 0.015921 |
| 10 | wavelet.HHH_firstorder_Mean_t1_3 | 0.506075 |
|  |  |  |

**Table S7 T2_R1 radiomics features selected for T2_R1_Score model construction using LASSO method.**

| index | Feature Name | Weighted. |
| --- | --- | --- |
| 0 | (Intercept) | -0.15376 |
| 1 | original_glszm_ZonePercentage_t2_1 | 0.102985 |

**Table S8 T2_R2 radiomics features selected for T2_R2_Score model construction using LASSO method.**

| index | Feature Name | Weighted. |
| --- | --- | --- |
| 0 | (Intercept) | -0.1608 |
| 1 | original_glszm_GrayLevelNonUniformityNormalized_t2_2 | -0.04284 |
| 2 | original_glszm_GrayLevelVariance_t2_2 | 2.26E-13 |
| 3 | original_glszm_SizeZoneNonUniformity_t2_2 | 0.33426 |
| 4 | log.sigma.4.0.mm.3D_ngtdm_Strength_t2_2 | -0.13341 |
| 5 | wavelet.LLH_glszm_SmallAreaHighGrayLevelEmphasis_t2_2 | 0.245451 |
| 6 | wavelet.LHL_firstorder_Kurtosis_t2_2 | 0.249565 |
| 7 | wavelet.LHH_gldm_DependenceVariance_t2_2 | 0.104132 |
| 8 | wavelet.HLL_glszm_SizeZoneNonUniformity_t2_2 | 0.023586 |
| 9 | wavelet.HLH_glcm_JointAverage_t2_2 | -0.35864 |
| 10 | wavelet.HLH_glcm_SumAverage_t2_2 | -4.79E-13 |
| 11 | wavelet.HHL_firstorder_Skewness_t2_2 | -0.24219 |
| 12 | wavelet.HHL_glrlm_HighGrayLevelRunEmphasis_t2_2 | 0.302388 |

**Table S9 T2_R3 radiomics features selected for T2_R3_Score model construction using LASSO method.**

| index | Feature Name | Weighted. |
| --- | --- | --- |
| 0 | (Intercept) | -0.14367 |
| 1 | log.sigma.5.0.mm.3D_glszm_SizeZoneNonUniformity_t2_3 | 0.114107 |
| 2 | log.sigma.2.0.mm.3D_firstorder_Kurtosis_t2_3 | 0.141781 |
| 3 | wavelet.HLH_glszm_SizeZoneNonUniformity_t2_3 | 0.043262 |
| 4 | wavelet.LLH_firstorder_Skewness_t2_3 | -0.07396 |
| 5 | log.sigma.3.0.mm.3D_glszm_SmallAreaEmphasis_t2_3 | 0.081241 |
| 6 | wavelet.HLL_firstorder_Kurtosis_t2_3 | 0.16339 |
| 7 | wavelet.LLL_glcm_Idn_t2_3 | -0.31739 |

**Table S10 T2f_R1 radiomics features selected for T2f_R1_Score model construction using LASSO method.**

| index | Feature Name | Weighted. |
| --- | --- | --- |
| 0 | (Intercept) | -0.18028 |
| 1 | log.sigma.3.0.mm.3D_glszm_GrayLevelNonUniformityNormalized_t2f_1 | -0.32554 |
| 2 | log.sigma.5.0.mm.3D_firstorder_RootMeanSquared_t2f_1 | 0.438869 |
| 3 | wavelet.LLL_firstorder_Maximum_t2f_1 | 0.331963 |
| 4 | wavelet.LLH_gldm_DependenceVariance_t2f_1 | -0.42224 |
| 5 | wavelet.LHL_glszm_SmallAreaEmphasis_t2f_1 | 0.050108 |
| 6 | wavelet.HHL_firstorder_RobustMeanAbsoluteDeviation_t2f_1 | -1.17634 |
| 7 | wavelet.LLL_firstorder_Median_t2f_1 | -0.47293 |
| 8 | wavelet.LHH_glcm_MCC_t2f_1 | -0.22854 |
| 9 | wavelet.HLL_glszm_SmallAreaHighGrayLevelEmphasis_t2f_1 | -0.19464 |
| 10 | original_gldm_SmallDependenceHighGrayLevelEmphasis_t2f_1 | 0.09839 |
| 11 | wavelet.LHH_glszm_GrayLevelVariance_t2f_1 | 0.357709 |
| 12 | wavelet.HHL_glcm_SumEntropy_t2f_1 | -0.26242 |
| 13 | log.sigma.3.0.mm.3D_glcm_Imc1_t2f_1 | 0.465796 |
| 14 | wavelet.LLL_glszm_SizeZoneNonUniformityNormalized_t2f_1 | -0.24463 |
| 15 | log.sigma.5.0.mm.3D_firstorder_Minimum_t2f_1 | 0.402182 |

**Table S11 T2f_R2 radiomics features selected for T2f_R2_Score model construction using LASSO method.**

| index | Feature Name | Weighted. |
| --- | --- | --- |
| 0 | (Intercept) | -0.10391 |
| 1 | log.sigma.2.0.mm.3D_ngtdm_Busyness_t2f_2 | 0.025914 |
| 2 | log.sigma.2.0.mm.3D_gldm_DependenceNonUniformityNormalized_t2f_2 | -0.09956 |
| 3 | log.sigma.3.0.mm.3D_glszm_SizeZoneNonUniformity_t2f_2 | 0.254416 |
| 4 | wavelet.LHH_glcm_SumEntropy_t2f_2 | 0.163825 |
| 5 | wavelet.HLL_glcm_SumEntropy_t2f_2 | 0.085023 |
| 6 | wavelet.HHL_firstorder_Skewness_t2f_2 | 0.229175 |

**Table S12 T2f_R3 radiomics features selected for T2f_R3_Score model construction using LASSO method.**

| index | Feature Name | Weighted. |
| --- | --- | --- |
| 0 | (Intercept) | -0.18864 |
| 1 | wavelet.LLL_glrlm_ShortRunLowGrayLevelEmphasis_t2f_3 | 0.040461 |
| 2 | log.sigma.5.0.mm.3D_firstorder_Minimum_t2f_3 | 0.408094 |
| 3 | wavelet.LLH_glszm_SizeZoneNonUniformity_t2f_3 | 0.064809 |
| 4 | wavelet.HHL_firstorder_Median_t2f_3 | -0.18888 |
| 5 | original_firstorder_Median_t2f_3 | -0.19452 |
| 6 | wavelet.HHH_glcm_SumEntropy_t2f_3 | 0.154295 |
| 7 | wavelet.LLL_firstorder_Uniformity_t2f_3 | -0.07976 |
| 8 | log.sigma.5.0.mm.3D_firstorder_Variance_t2f_3 | -0.02844 |
| 9 | wavelet.HLL_glszm_SmallAreaEmphasis_t2f_3 | 0.104929 |
| 10 | log.sigma.4.0.mm.3D_glszm_SizeZoneNonUniformity_t2f_3 | 0.19657 |
| 11 | wavelet.LLL_glszm_LowGrayLevelZoneEmphasis_t2f_3 | -0.15639 |
| 12 | wavelet.HLH_glszm_SmallAreaHighGrayLevelEmphasis_t2f_3 | 0.198853 |
| 13 | wavelet.HLL_firstorder_Mean_t2f_3 | 0.260666 |
| 14 | wavelet.HHL_glrlm_LowGrayLevelRunEmphasis_t2f_3 | 0.377224 |
| 15 | original_glszm_SizeZoneNonUniformity_t2f_3 | 0.442835 |
|  |  |  |

**Table S13 Clinical model construction using Logistic Regression method**

| Index | Feature Name | *β-*value. | *p*-value |
| --- | --- | --- | --- |
| 0 | Intercept | 0.572 | 0.134 |
| 1 | Gender | -0.648 | 0.119 |
| 2 | Deep.WM.Invasion | -0.648 | 0.119 |

Deep.WM.Invasion: Deep White Matter Invasion.

**Table S14 Detailed ROC-related metrics for all constructed models.**

| Models | | AUC(95%CI) | Accuracy | Sensitivity | Specificity |
| --- | --- | --- | --- | --- | --- |
| T1c_R1_Score | train | 0.702 (0.598-0.807) | 0.7 | 0.522 | 0.852 |
|  | test1 | 0.528 (0.342-0.714) | 0.651 | 0.4 | 0.87 |
|  | test2 | 0.544 (0.343-0.745) | 0.675 | 0.357 | 0.846 |
| T1c_R2_Score | train | 0.841 (0.763-0.918) | 0.79 | 0.674 | 0.889 |
|  | test1 | 0.517 (0.338-0.697) | 0.581 | 0.7 | 0.478 |
|  | test2 | 0.613 (0.424-0.801) | 0.625 | 0.643 | 0.615 |
| T1c_R3_Score | train | 0.778 (0.687-0.869) | 0.74 | 0.652 | 0.815 |
|  | test1 | 0.589 (0.409-0.769) | 0.628 | 0.6 | 0.652 |
|  | test2 | 0.679 (0.508-0.849) | 0.625 | 0.857 | 0.5 |
| T1_R1_Score | train | 0.875 (0.807-0.943) | 0.82 | 0.761 | 0.87 |
|  | test1 | 0.811 (0.676-0.946) | 0.791 | 0.85 | 0.739 |
|  | test2 | 0.58 (0.372-0.787) | 0.725 | 0.5 | 0.846 |
| T1_R2_Score | train | 0.924 (0.871-0.976) | 0.87 | 0.978 | 0.778 |
|  | test1 | 0.759 (0.611-0.906) | 0.721 | 0.5 | 0.913 |
|  | test2 | 0.692 (0.562-0.823) | 0.625 | 0.857 | 0.5 |
| T1_R3_Score | train | 0.777 (0.684-0.869) | 0.77 | 0.696 | 0.833 |
|  | test1 | 0.533 (0.349-0.716) | 0.628 | 0.5 | 0.739 |
|  | test2 | 0.602 (0.404-0.799) | 0.7 | 0.571 | 0.769 |
| T2_R1_Score | train | 0.634 (0.521-0.748) | 0.67 | 0.391 | 0.907 |
|  | test1 | 0.616 (0.441-0.791) | 0.674 | 0.85 | 0.522 |
|  | test2 | 0.588 (0.401-0.775) | 0.55 | 0.929 | 0.346 |
| T2_R2_Score | train | 0.798 (0.709-0.887) | 0.75 | 0.652 | 0.833 |
|  | test1 | 0.611 (0.436-0.786) | 0.651 | 0.8 | 0.522 |
|  | test2 | 0.621 (0.423-0.819) | 0.65 | 0.571 | 0.692 |
| T2_R3_Score | train | 0.717 (0.615-0.82) | 0.71 | 0.587 | 0.815 |
|  | test1 | 0.528 (0.35-0.707) | 0.581 | 0.75 | 0.435 |
|  | test2 | 0.651 (0.469-0.833) | 0.6 | 0.857 | 0.462 |
| T2f_R1_Score | train | 0.825 (0.745-0.906) | 0.77 | 0.826 | 0.722 |
|  | test1 | 0.583 (0.405-0.76) | 0.628 | 0.65 | 0.609 |
|  | test2 | 0.558 (0.354-0.761) | 0.7 | 0.5 | 0.808 |
| T2f_R2_Score | train | 0.718 (0.614-0.822) | 0.73 | 0.435 | 0.981 |
|  | test1 | 0.667 (0.504-0.831) | 0.628 | 1 | 0.304 |
|  | test2 | 0.602 (0.411-0.793) | 0.7 | 0.571 | 0.769 |
| T2f_R3_Score | train | 0.815 (0.732-0.899) | 0.75 | 0.783 | 0.722 |
|  | test1 | 0.465 (0.284-0.646) | 0.535 | 1 | 0.13 |
|  | test2 | 0.569 (0.374-0.763) | 0.65 | 0.5 | 0.731 |

Youden criterion: The best operating point of the ROC was chosen at the point whose Youden index (Sensitivity+Specificity-1) is maximal.

**Table S15 T1cScore model construction using Logistic Regression method**

| Index | Feature Name | *β-*value. | *p*-value |
| --- | --- | --- | --- |
| 0 | Intercept | -6.926 | 0.000 |
| 1 | T1c_R1_Score | 6.971 | 0.013 |
| 2 | T1c_R2_Score | 3.753 | 0.002 |
| 3 | T1c_R3_Score | 3.940 | 0.013 |

**Table S16 T1Score model construction using Logistic Regression method**

| Index | Feature Name | *β-*value. | *p*-value |
| --- | --- | --- | --- |
| 0 | Intercept | -11.670 | 0.000 |
| 1 | T1_R1_Score | 10.554 | 0.000 |
| 2 | T1_R2_Score | 9.264 | 0.000 |
| 3 | T1_R3_Score | 4.314 | 0.063 |

**Table S17 T2Score model construction using Logistic Regression method**

| Index | Feature Name | *β-*value. | *p*-value |
| --- | --- | --- | --- |
| 0 | Intercept | -14.214 | 0.005 |
| 1 | T2_R1_Score | 21.784 | 0.046 |
| 2 | T2_R2_Score | 5.880 | 0.001 |
| 3 | T2_R3_Score | 2.872 | 0.243 |

**Table S18 T2fScore model construction using Logistic Regression method**

| Index | Feature Name | *β-*value. | *p*-value |
| --- | --- | --- | --- |
| 0 | Intercept | -6.194 | 0.000 |
| 1 | T2f_R1_Score | 4.351 | 0.001 |
| 2 | T2f_R2_Score | 4.453 | 0.114 |
| 3 | T2f_R3_Score | 4.163 | 0.005 |

**Table S19 R1Score model construction using Logistic Regression method**

| Index | Feature Name | *β-*value. | *p*-value |
| --- | --- | --- | --- |
| 0 | Intercept | 3.462 | 0.606 |
| 1 | T1c_R1_Score | 3.577 | 0.300 |
| 2 | T1_R1_Score | 5.396 | 0.000 |
| 3 | T2_R1_Score | -21.107 | 0.199 |
| 4 | T2f_R1_Score | 4.274 | 0.056 |

**Table S20 R2Score model construction using Logistic Regression method**

| Index | Feature Name | *β-*value. | *p*-value |
| --- | --- | --- | --- |
| 0 | Intercept | 1.828 | 0.001 |
| 1 | T1c_R2_Score | 1.422 | 0.114 |
| 2 | T1_R2_Score | 1.688 | 0.000 |
| 3 | T2_R2_Score | 2.101 | 0.506 |
| 4 | T2f_R2_Score | 3.910 | 0.548 |

**Table S21 R3Score model construction using Logistic Regression method**

| Index | Feature Name | *β-*value. | *p*-value |
| --- | --- | --- | --- |
| 0 | Intercept | -6.023 | 0.000 |
| 1 | T1c_R3_Score | 2.483 | 0.155 |
| 2 | T1_R3_Score | 3.323 | 0.033 |
| 3 | T2_R3_Score | 1.864 | 0.500 |
| 4 | T2f_R3_Score | 5.077 | 0.000 |

**Table S22 CombRadScore model construction using Logistic Regression method**

| Index | Feature Name | *β-*value. | *p*-value |
| --- | --- | --- | --- |
| 0 | Intercept | -160.40 | 0.102 |
| 1 | T1c_R1_Score | 37.60 | 0.122 |
| 2 | T1c_R2_Score | -29.83 | 0135 |
| 3 | T1c_R3_Score | 17.71 | 0.157 |
| 4 | T1_R1_Score | 86.36 | 0.110 |
| 5 | T1_R2_Score | 47.43 | 0.105 |
| 6 | T1_R3_Score | 22.50 | 0.095 |
| 7 | T2_R2_Score | 54.38 | 0.117 |
| 8 | T2_R3_Score | -32.48 | 0.138 |
| 9 | T2f_R1_Score | -40.77 | 0.117 |
| 10 | T2f_R2_Score | 28.62 | 0.170 |
| 11 | T2f_R3_Score | 37.29 | 0.110 |

**Table S23 Rad+Clinical model construction using Logistic Regression method**

| Index | Feature Name | *β-*value. | *p*-value |
| --- | --- | --- | --- |
| 0 | Intercept | -3.946 | 0.012 |
| 1 | Gender | -0.829 | 0.569 |
| 2 | Deep.WM.Invasion | -0.269 | 0.843 |
| 3 | CombRadScore | 9.714 | 0.000 |

Deep.WM.Invasion: Deep White Matter Invasion.

**Table S24 Kendall correlation analysis among the variables using in the Rad+Clinical model**

| - | CombRadScore | Gender. | Deep.WM.Invasion |
| --- | --- | --- | --- |
| CombRadScore | *-* | *τ*=-0.163, *p*=0.059 | *τ*=-0.098, *p*=0.257 |
| Gender. | *τ*=-0.163, *p*=0.059 | - | *τ*=0.102, *p*=0.308 |
| Deep.WM.Invasion | *τ*=-0.098, *p*=0.257 | *τ*=0.102, *p*=0.308 | - |
